# A Tumor-Responsive Enzymatic Cascade System Inducing pH-Activable Metabolic Starvation and H_2_O_2_-Induced Apoptosis

**DOI:** 10.34133/bmr.0380

**Published:** 2026-06-09

**Authors:** Junyoung Jung, Jae Hun Lee, Seoungkyun Kim, Kiyoon Kwon, Giyoong Tae, Inchan Kwon

**Affiliations:** Department of Materials Science and Engineering, Gwangju Institute of Science and Technology (GIST), Gwangju 61005, Republic of Korea.

## Abstract

Conventional tumor-targeted therapies primarily focus on drug delivery systems exploiting the tumor microenvironment for controlled drug release. While prodrug strategies activate small-molecule drugs in tumors, they rely on single-step chemical conversions and lack sustained tumor-selective activity. Here, a novel strategy of tumor-responsive, enzymatic cascade therapy is introduced by co-delivering arginine decarboxylase (RDC) and diamine oxidase (DAO) into the tumor site. Unlike traditional arginine depletion strategies, RDC remains inactive at physiological pH and is selectively activated under acidic tumor conditions, ensuring localized metabolic starvation while minimizing systemic toxicity. To overcome a key limitation of RDC—namely, the accumulation of toxic agmatine—RDC is co-delivered with DAO using a chitosan-functionalized Pluronic-based nanocarrier (RDC/DAO@NC), enabling tumor-specific conversion of agmatine into hydrogen peroxide (H_2_O_2_). This pH-sensitive cascade reaction is expected to limit agmatine accumulation in normal tissues while amplifying RDC-mediated antitumor effects through oxidative-stress-induced apoptosis. In vitro, RDC/DAO@NC suppresses tumor cell proliferation through arginine depletion and induces apoptosis via H_2_O_2_ generation. In vivo, RDC/DAO@NC substantially enhances tumor accumulation and therapeutic efficacy compared to free enzymes. This study provides the first in vivo validation of RDC-based cancer therapy, suggesting its potential as a tumor-selective strategy integrating enzyme activation in the tumor microenvironment, metabolic starvation, and reactive-oxygen-species-induced apoptosis in cancer treatment.

## Introduction

The tumor microenvironment (TME) is characterized by unique physiological conditions, including hypoxia, slightly acidic pH, oxidative stress, and nutrient depletion, which distinguish it from normal tissues [1]. These hallmarks have been extensively exploited in the design of tumor-responsive drug delivery systems, where anticancer drugs are released through the cleavage of linkers or the disassembly of nanostructures within the TME [2,3]. For example, hypoxia-sensitive groups such as quinone, nitroaromatic, and azobenzene derivatives have been utilized for tumor-specific drug delivery and tumor imaging [4,5]. Acidic pH has been explored for developing pH-responsive polymeric nanocarriers, which enable controlled drug release through volume expansion, pH-sensitive cross-linkers, and hydrophobic-to-hydrophilic transitions [6,7]. Additionally, pH-responsive DNA aptamers, which selectively bind to target proteins in cancer cells under acidic conditions, have been explored as promising tools for precision cancer therapy [8]. Apart from pH and hypoxia, tumor-associated biomolecules, such as peptides and enzymes overexpressed in the TME, have emerged as promising tools for anticancer therapy. Based on the elevated intracellular levels of glutathione in tumor cells compared to normal tissues [9], various glutathione-responsive drug delivery systems have been developed, particularly through prodrug molecules containing disulfide bonds or α, β-unsaturated carbonyl groups [10]. The dysregulated overexpression and hyperactivation of matrix metalloproteinases in the TME, which contribute to tumor progression, invasion, and metastasis [11], have also been applied for the development of stimuli-responsive drug delivery systems and targeted cancer therapies [12]. In addition, prodrug-based strategies have been developed to achieve tumor-specific drug activation [13,14]. Prodrugs are chemically modified small molecules that remain inactive in circulation and undergo chemical conversion into active drugs in response to tumor-specific biochemical cues, such as pH, hypoxia, or enzyme activity [13,14]. While effective, prodrugs rely on passive cleavage mechanisms and do not sustain tumor-selective activity after activation, which can limit their therapeutic impact. Despite these advancements in tumor-targeted drug delivery, a fundamental limitation remains: These systems control drug release, but the active drug itself does not necessarily exhibit tumor specificity. Therefore, a new strategy is needed, where the therapeutic agent itself, rather than just the delivery system, is tumor responsive. In addition, active-targeting strategies employing ligand–receptor interactions have been widely explored to enhance tumor-specific delivery of nanocarriers [15,16].

Enzyme cascade-based nanotherapeutic systems have been widely developed to enhance anticancer efficacy by co-delivering multiple enzymes that induce metabolic starvation and/or reactive oxygen species (ROS) generation within tumor tissues [17–19]. In these systems, tumor selectivity is primarily achieved through nanocarrier-mediated delivery and local accumulation, often in combination with auxiliary triggers such as photothermal activation or redox-responsive processes. However, in most reported systems, the therapeutic enzymes themselves remain catalytically active under physiological conditions, and their activity is not intrinsically regulated by tumor-specific physicochemical cues.

Here, we introduce a tumor-responsive enzymatic cascade therapy, in which pH-dependent arginine decarboxylase (RDC) functions as a tumor-selective therapeutic enzyme. RDC undergoes a reversible transition from an inactive dimer to an active decamer under acidic conditions, where oligomerization enables proper active-site organization required for catalysis [20,21]. Cancer progression is driven by dysregulated metabolic processes that support uncontrolled cell proliferation. A key aspect of this altered metabolism is an increased demand for nutrients, particularly amino acids, which are essential for biosynthetic pathways like protein and polyamine production, thereby providing critical resources for cancer cell growth [22]. Unlike normal cells, which can synthesize these molecules, many cancer types, such as those affecting the pancreas, liver, and lungs, cannot produce certain amino acids, such as arginine, due to defective enzymes like argininosuccinate synthetase (ASS) and argininosuccinate lyase [23], making them dependent on external sources of arginine. This metabolic vulnerability provides an opportunity for therapeutic intervention through arginine depletion, which has shown promise in selectively inducing metabolic stress in cancer cells and inhibiting their proliferation [24]. Arginine depletion works by cutting off the supply of this critical nutrient, leading to selective cancer cell death without affecting normal cells that retain the ability to synthesize arginine via the urea cycle [25]. This approach has gained significant interest as an anticancer strategy, particularly through the use of arginine-depleting enzymes [26]. For this purpose, arginine deiminase, which catalyzes the hydrolysis of arginine into citrulline and ammonia, and arginase (Arg), which converts arginine into ornithine and urea, have been extensively studied [27]. However, these enzymes lack tumor specificity, remain catalytically active at physiological pH, and may cause systemic toxicity in normal tissues. In contrast, RDC exhibits an intrinsic pH-dependent switch: It is inactive at physiological pH but becomes catalytically active under the acidic conditions of the TME by undergoing structural reorganization into a decamer composed of 5 dimers [20,28]. This unique pH-responsive behavior makes RDC a promising candidate for selective arginine depletion in tumor tissues while minimizing toxicity in normal tissues.

Despite these advantages, arginine-deprivation therapy using RDC remains a significant challenge due to the inherent limitation of the short half-life and its product, agmatine, which may exert cytotoxic effects and cannot be converted back to arginine under physiological conditions [27]. Here, to overcome the limitations of RDC and further enhance the therapeutic efficacy, we implemented a cascade enzyme reaction strategy by co-delivering RDC and diamine oxidase (DAO). DAO catalyzes the oxidative degradation of agmatine, the product of RDC, into hydrogen peroxide (H_2_O_2_), introducing a ROS-mediated cytotoxic mechanism that synergizes with arginine depletion [29]. This 2-pronged approach not only enhances antitumor efficacy but also mitigates the potential off-target effects of agmatine accumulation.

For effective enzyme co-delivery, we utilized a chitosan-functionalized, Pluronic-based nanocarrier (NC) that encapsulates enzymes with high loading efficiency through a simple, temperature-responsive encapsulation process. The NC serves as a tumor-targeting platform that enhances stability and promotes controlled release of loaded enzymes [30]. The NC also functions as a nanoreactor that amplifies the enzymatic cascade reaction between loaded enzymes, facilitating the conversion of intermediates into final products for enhanced efficacy at the tumor site [31,32]. This tumor-responsive enzyme therapy represents a potential advancement over conventional enzyme-based cancer treatments.

**Fig. 1.**
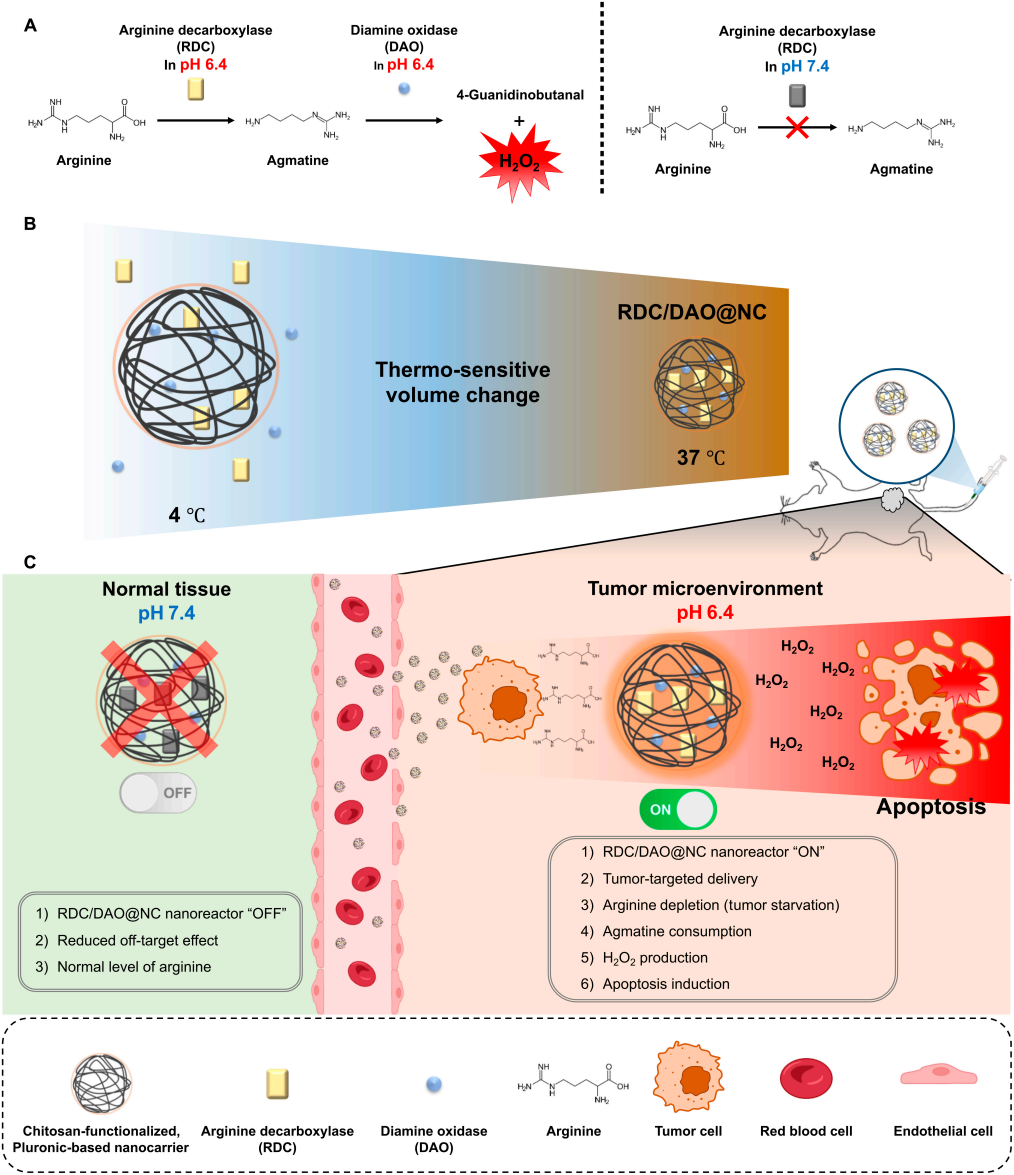
Schematic representation of arginine decarboxylase (RDC)- and diamine oxidase (DAO)-loaded nanocarrier (RDC/DAO@NC) administration for cancer-specific therapeutics. (A) pH-dependent activation of the RDC/DAO enzymatic cascade. (B) Thermo-responsive enzyme loading into NCs. (C) Tumor-specific activation of the nanoreactor (RDC/DAO@NC).

Through this study, we provide the first in vivo demonstration of RDC’s antitumor efficacy with minimal systemic toxicity and introduce a synergistic enzyme cascade system for enhanced cancer treatment. By integrating tumor-environment responsiveness, metabolic depletion, and ROS-mediated cytotoxicity, this platform offers a new strategy for precision cancer therapy with tumor-activated therapeutic enzymes (Fig. 1).

## Materials and Methods

### Materials

Information on the materials used in this study is provided in the Supplementary Materials.

### Preparation and characterization of RDC- and DAO-loaded NC

RDC was prepared and purified as previously reported [28], and the chitosan-conjugated Pluronic NC was prepared as described previously [30] and in Sections S1 and S2. RDC and DAO were encapsulated using the thermo-responsive volumetric transition of NC, with enzyme loading at 4 °C followed by contraction at 37 °C. Unencapsulated enzymes were removed by centrifugal filtration, and RDC alone was loaded to prepare RDC@NC. Hydrodynamic diameter, polydispersity, and zeta potential were measured by dynamic light scattering, while morphology was measured by transmission electron microscopy. Encapsulation efficiency was quantified using Alexa 488-labeled enzymes, and enzyme release was evaluated by fluorescence- and activity-based assays. Detailed procedures are provided in Section S3.

### Cascade reaction of free enzyme and co-encapsulated enzyme inside NCs

The cascade reaction involving RDC and DAO was set up with RDC at a constant concentration (0.1 mg/ml) while varying the DAO concentrations to achieve RDC:DAO mass ratios of 1:1, 1:2, and 1:5, respectively. For reactions using free enzymes, RDC and DAO were directly added to the mixtures. For encapsulation experiments, RDC (10 mg/ml, 40 μl) and DAO (10, 20, or 50 mg/ml, 40 μl) were encapsulated into 100 μl of NC (10 mg/ml).

Arginine was added to achieve a final concentration of 10 mM. The reaction solutions, totaling 200 μl, were incubated at 37 °C at pH levels of either 6.4 or 7.4. The production of hydrogen peroxide was quantified using the horseradish peroxidase (HRP)/homovanillic acid system, as described in Section S3.4.

### In vitro antitumor activity of RDC- and DAO-loaded NC

To evaluate the anticancer effects of RDC and DAO at the cellular level, Dulbecco’s Modified Eagle Medium adjusted to pH 6.4 using HCl was utilized to mimic the acidic TME. Mouse squamous cell carcinoma 7 (SCC7) cells or human breast adenocarcinoma MCF7 cells were seeded into a 96-well plate at a density of 3,000 cells per well and allowed to adhere for 24 h. The cells were then treated with NC, RDC, DAO, RDC/DAO, and RDC- and DAO-loaded NC (RDC/DAO@NC; 0.25 mg/ml of NC, 0.1 mg/ml of RDC, and 0.1 mg/ml of DAO) and incubated for 3 d. After incubation, cells were washed with phosphate-buffered saline (PBS) and subjected to a Live/Dead assay using calcein acetoxymethyl and propidium iodide (PI).

Simultaneously, cells cultured under the same conditions in a separate 96-well plate were detached using trypsin without washing, and the total number of cells in each well was counted using a hemocytometer. The number of live and dead cells was quantified.

To further analyze cascade-induced oxidative stress and apoptosis, dihydroethidium (DHE) staining and Annexin V/PI staining were performed. SCC7 cells were seeded into a 96-well plate at a density of 3,000 cells per well using pH 6.4 Dulbecco’s Modified Eagle Medium. After 24 h of incubation, the cells were treated with NC, RDC, DAO, RDC/DAO, or RDC/DAO@NC (2.5 mg/ml of NC, 1 mg/ml of RDC, and 1 mg/ml of DAO). Following a 24-h incubation, the cells were washed once with PBS and stained with DHE or with Alexa Fluor 488–Annexin V and PI according to the manufacturer’s protocol. Stained cells were imaged using fluorescence microscopy, and DHE-positive areas, Hoechst-stained nuclei, Annexin V-positive cells, and PI-positive cells were quantified using ImageJ software.

### Animal experiment

All animal experiments were conducted with the approval of the Institutional Animal Care and Use Committee at the Gwangju Institute of Science and Technology (GIST-2023-046). Six-week-old male nude mice (20 g) were maintained under a 12-h light/dark cycle with free access to food and water.

### Biodistribution and pharmacokinetics of RDC/DAO@NC

Six-week-old nude mice were subcutaneously injected with 1.5 × 10^6^ SCC7 cells, and 13 d postinoculation, mice received intravenous injections of Alexa 680-labeled RDC/DAO or Alexa 680-labeled RDC/DAO@NC (NC: 100 mg/kg, Alexa 680–RDC: 40 mg/kg, and DAO: 40 mg/kg). At 1, 2, and 3 d following injection, the major organs (heart, spleen, lung, liver, and kidneys) and tumor tissue were excised, and fluorescence signals were measured using FOBI (Fluorescence In Vivo Imaging System; NeoScience, Seoul, Korea).

For pharmacokinetic analysis, Cy5.5-labeled RDC/DAO or Cy5.5-labeled RDC/DAO@NC was intravenously injected into healthy nude mice at identical doses. Blood samples were collected from the retro-orbital sinus at predetermined time points (0.5, 1, 2, 4, 6, 12, and 24 h). The collected blood samples were centrifuged at 1,500×*g* for 10 min at 4 °C, and the resulting plasma was used to determine circulation profiles.

### In vivo antitumor efficacy and biocompatibility of RDC/DAO@NC

SCC7 cells (1.5 × 10^6^) were subcutaneously injected into 6-week-old male nude mice. Once the tumor volumes reached ~50 mm^3^, the mice were randomly divided into 4 groups (*n* = 5). The groups were administered the assigned treatments every 3 d for a total of 4 injections: control (PBS), RDC/DAO, RDC@NC, or RDC/DAO@NC (NC: 100 mg/kg, RDC: 40 mg/kg, and DAO: 40 mg/kg). Tumor size was measured using a vernier caliper, and tumor volume was calculated as 0.5 × (Length) × (Width)^2^.

Three days after the final injection, blood serum, major organs (heart, liver, lungs, spleen, and kidneys), and tumor tissues were harvested for analysis. Tumor weights were recorded, and serum samples were analyzed to evaluate biocompatibility by measuring liver function markers, aspartate aminotransferase (AST) and alanine aminotransferase (ALT), and kidney function markers, blood urea nitrogen (BUN) and creatinine. The collected organs and tumor tissues were fixed in 4% formaldehyde, dehydrated using graded ethanol and xylene, and embedded in paraffin. The paraffin-embedded tissues were sectioned and stained with hematoxylin and eosin (H&E).

A terminal deoxynucleotidyl transferase dUTP nick end labeling (TUNEL) assay was performed on tumor sections to identify apoptotic cells. Additionally, Ki-67 immunostaining was conducted using HRP and 3,3′-diaminobenzidine tetrahydrochloride salt to assess tumor cell proliferation. To evaluate ROS generation in tumor tissues, freshly isolated tumors were embedded in optimal cutting temperature compound without fixation, cryosectioned, and subjected to DHE staining.

To quantify intratumoral arginine levels, the tumor tissue was excised 3 d after the final injection. Approximately 200 mg of tumor tissue was placed in 1 ml of PBS containing 1 mM EDTA and homogenized on ice using a homogenizer (T25 digital Ultra-Turrax; IKA, Staufen, Germany) at 12,400 rpm for 1 min, followed by incubation in a 95 °C water bath for 10 min to denature all enzymes. The homogenates were centrifuged at 14,000×*g* for 10 min at 4 °C, and the supernatants were collected and lyophilized. Lyophilized samples were stored at –20 °C and reconstituted in 60 μl of Sample Diluent supplied with the kit. Intratumoral arginine levels were measured using the L-arginine competitive enzyme immunoassay/enzyme-linked immunosorbent assay kit according to the manufacturer’s protocol.

To investigate apoptosis-related signaling, Western blot analysis of caspase-3 and cleaved caspase-3 was performed. Tumor tissues (~50 mg) were homogenized in radioimmunoprecipitation assay buffer containing protease inhibitor using a homogenizer on ice (12,400 rpm, 10 s on/10 s off, 3 cycles), followed by incubation on ice for 10 min and centrifugation at 14,000×*g* for 10 min at 4 °C. Protein concentrations were determined using a bicinchoninic acid assay, and 50 μg of total protein per sample was loaded onto 4% to 12% Bis–Tris gels for sodium dodecyl sulfate–polyacrylamide gel electrophoresis. After electrophoresis, proteins were transferred to a polyvinylidene fluoride membrane and probed with primary antibodies against caspase-3, cleaved caspase-3, and glyceraldehyde-3-phosphate dehydrogenase, followed by HRP-conjugated secondary antibodies. Protein bands were visualized using a chemiluminescent substrate, and images were obtained using a chemiluminescence system (ChemiDoc; Bio-Rad, CA, USA).

### Statistical analysis

All statistical analyses were performed using analysis of variance or Student’s *t* test. Statistical significance in all data was defined as a *P* value of less than or equal to 0.05.

## Results and Discussion

### Preparation and characterization of RDC/DAO@NC

A chitosan-functionalized, Pluronic-based NC was prepared, as previously established and well characterized by our group [30]. The ^1^H nuclear magnetic resonance spectrum and TEM images confirmed that the NC was successfully reproduced in this study (Figs. S1 and S2). In addition, attenuated total reflectance–Fourier-transform infrared spectroscopy analysis was newly performed to further verify the presence of chitosan within the NC (Fig. S3). The characteristic chitosan-associated peaks, including the broad N–H/O–H stretching band at 3,200 to 3,600 cm^−1^ and the amide I band near 1,650 cm^−1^, were clearly observed in the NC [33,34].

RDC was prepared and purified as reported in [28]. Detailed information is provided in Section S2 and Fig. S4.

**Fig. 2.**
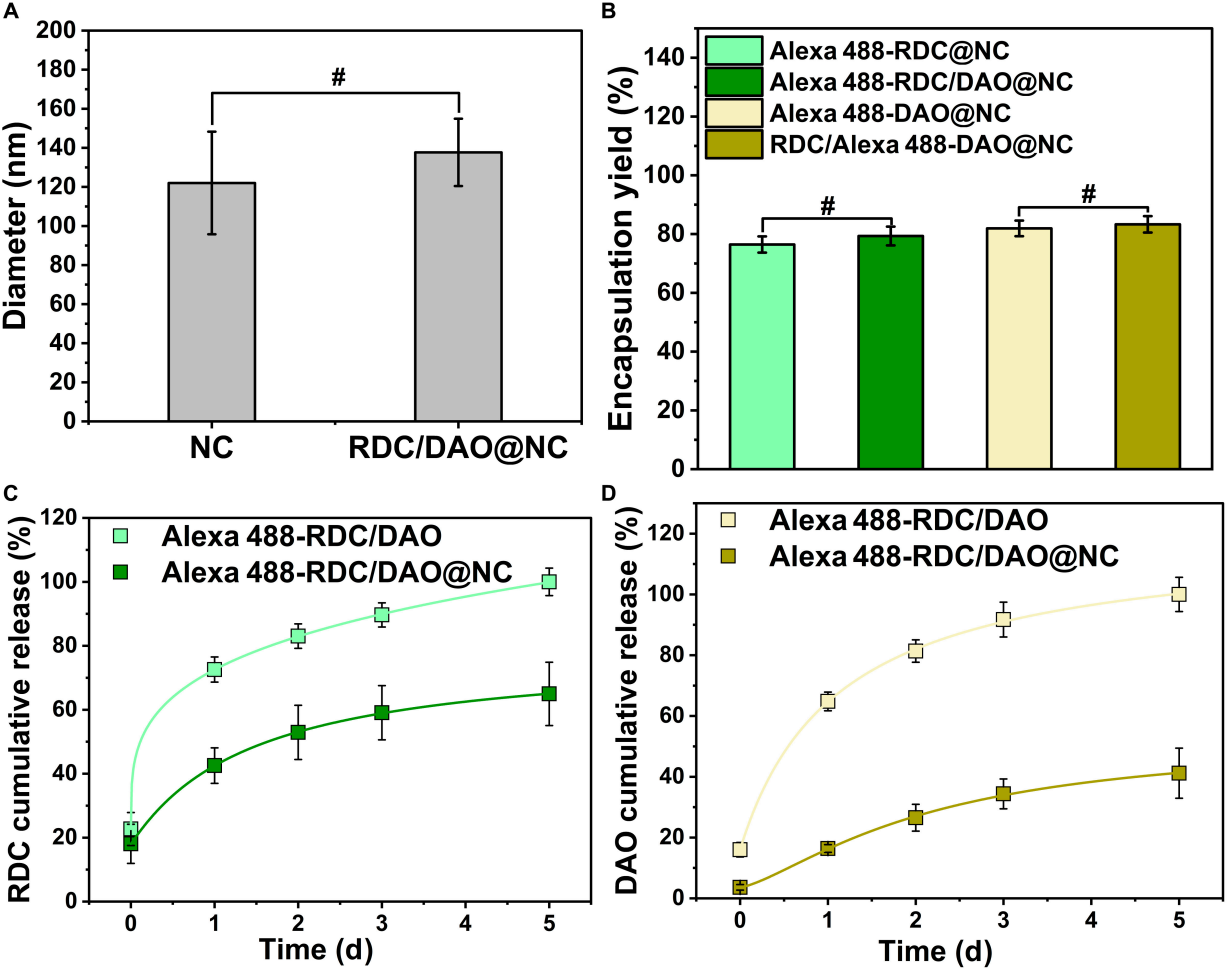
Characterization of RDC- and DAO-encapsulated NCs. (A) Dynamic light scattering (DLS) analysis comparing the size of NCs and RDC/DAO-loaded NCs (RDC/DAO@NC) (*n* = 3). (B) Encapsulation yield (%) of RDC and DAO in NCs (*n* = 3). (C) Release profile of RDC from NCs over time (*n* = 3). (D) Release profile of DAO from NCs over time (*n* = 3). Statistical significance: ^#^*P* > 0.05.

RDC and DAO were co-encapsulated into NC by co-incubating all at 4 °C overnight, followed by a temperature increase to 37 °C using the thermosensitive, large size change of NC [31,32]. The hydrodynamic diameter of NC at 37 °C was 122 ± 26 nm, and that of RDC/DAO@NC was 130 ± 14 nm. This indicates that there was no meaningful size change after enzyme loading (Fig. 2A and Table S1). Consistently, TEM imaging also revealed no noticeable changes in the spherical morphology or size of the NC after loading with RDC and DAO (Fig. S2). In addition, RDC/DAO@NC showed stable colloidal behavior under serum-containing conditions, with no appreciable aggregation for 3 d in PBS containing 10% FBS at 37 °C (Fig. S5).

The encapsulation efficiency of RDC and DAO was evaluated using Alexa 488 labeling. For individual encapsulation, RDC achieved a yield of 76.4 ± 2.8%, while DAO had a slightly higher yield of 81.9 ± 2.7%. In co-encapsulation, the encapsulation yields of RDC (79.4 ± 3.2%) and DAO (83.3 ± 2.8%) were comparable to those of single encapsulation (Fig. 2B). These findings confirm efficient encapsulation of both enzymes, with approximately 80% of each enzyme successfully encapsulated in all conditions. Importantly, co-encapsulation did not adversely affect encapsulation efficiency, as both RDC and DAO maintained high yields in the co-encapsulated setup. Furthermore, these results align with previously reported high loading efficiencies for proteins using the same type of NC [31,32]. Such high loading efficiency is not typically achieved in conventional nanocarrier systems, such as polymeric nanoparticles or liposomes [35,36]. A slight decrease in the zeta potential from 6.93 ± 1.69 mV to −2.29 ± 0.75 mV after RDC and DAO loading (Table S1) is presumably associated with the very high loading content of the enzymes in NC. A fraction of the enzymes may be present on the nanocarrier surface. However, the majority of enzymes were loaded inside the NC.

To quantify the release kinetics of enzymes from the NC, Alexa 488-labeled RDC was used, whereas the released amount of DAO was determined based on its enzymatic activity. Direct measurement of enzymatic activity for RDC was avoided because the addition of its substrate, arginine, would initiate a cascade reaction with DAO, complicating the quantification of RDC activity. Instead, fluorescence-based detection was employed to ensure accurate quantification. The release profiles of RDC and DAO showed that both RDC and DAO were released much more slowly from the NC when they were co-loaded compared to the free enzyme controls (Fig. 2C and D). Due to the size-dependent transport limitation of large proteins in the filtration-based setup, the apparent recovery of free enzymes may be underestimated. Therefore, the release profiles were interpreted mainly in a relative manner between groups. In the case of RDC, ~40% of RDC was released in the first day, with cumulative release reaching ~60% by day 5. DAO exhibited an even slower release rate than RDC, with ~20% released after 1 d and ~40% by day 5, confirming its gradual diffusion from the NC. The relatively slower release of DAO compared to RDC is likely attributed to its larger molecular size, as DAO has a ~175-kDa molecular weight, whereas RDC has a smaller molecular weight of ~85 kDa, facilitating its faster diffusion. These results demonstrate that controlled and sustained release of RDC and DAO was achieved using the NC. This further supports its function as a tumor-targeting carrier and an effective nanoreactor for facilitating the enzymatic cascade reaction.

### Enhanced cascade reaction between RDC and DAO in NC in vitro

**Fig. 3.**
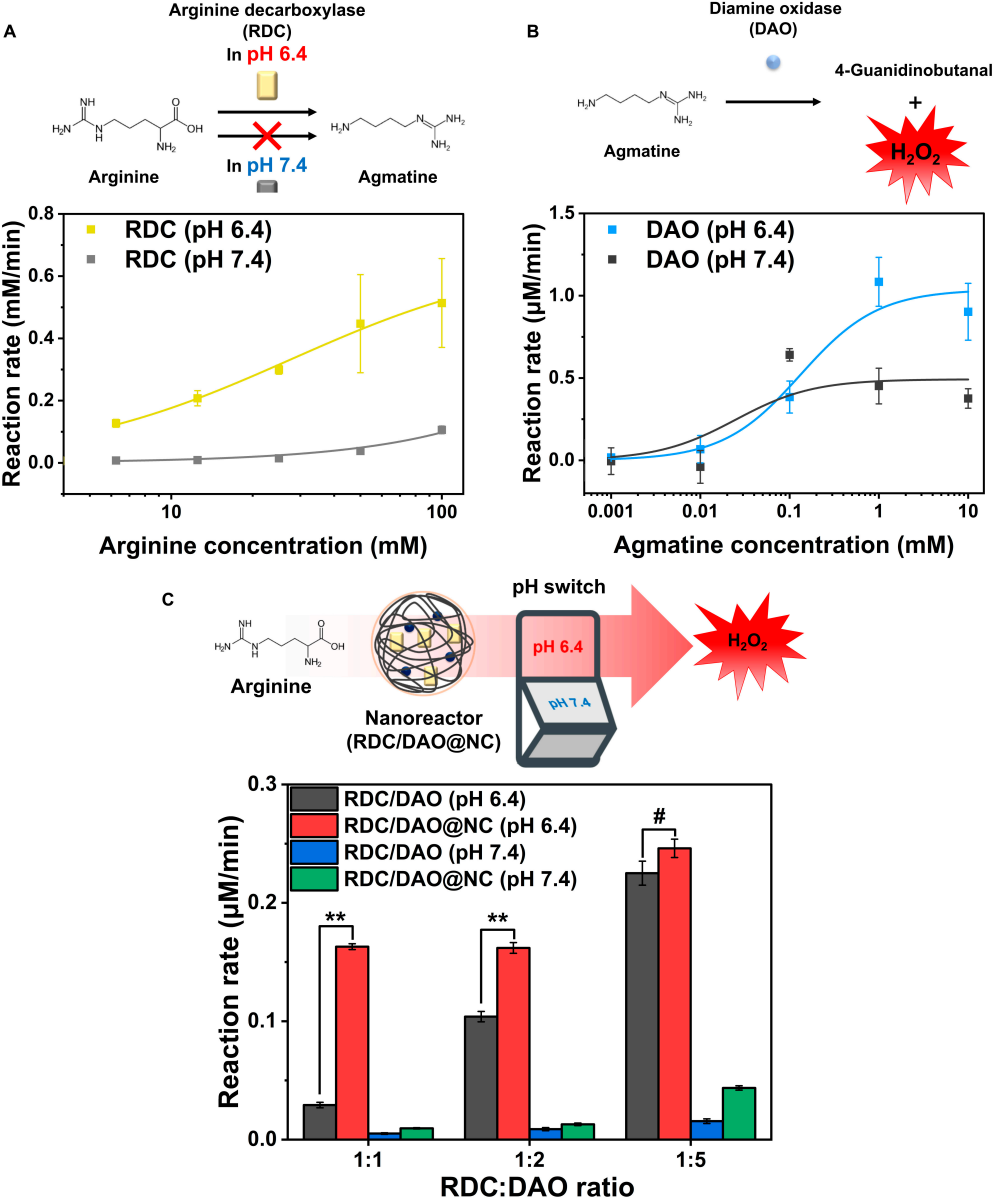
pH-dependent catalytic activity of RDC, DAO, and their cascade reaction. (A) Enzymatic activity of RDC at pH 6.4 and pH 7.4 as a function of arginine concentration (*n* = 3). (B) Enzymatic activity of DAO at pH 6.4 and pH 7.4 as a function of agmatine concentration (*n* = 3). (C) Cascade reaction rates of RDC and DAO at different RDC-to-DAO ratios under pH 6.4 and pH 7.4, in the presence or absence of the NC (*n* = 3). Statistical significance: ^#^*P* > 0.05, **P < 0.01.

The enzymatic activities of RDC and DAO were evaluated under 2 pH conditions (pH 6.4 and 7.4) and across different substrate concentrations (Fig. 3). Our results reveal that RDC exhibits markedly enhanced activity at the lower pH of 6.4 compared to pH 7.4 (Fig. 3A). This enhancement is particularly notable at specific concentrations of arginine, where RDC catalyzed the formation of agmatine. At concentrations of 6.25 and 12.5 mM arginine, the enzyme activity increased by 16-fold and 23-fold, respectively, demonstrating a substantial pH-dependent catalytic efficiency. Furthermore, this trend of increased enzymatic activity was consistently observed even as the concentration of arginine was elevated to 100 mM. According to the previous reports, RDC exists in an inactive dimeric form at physiological pH, whereas under acidic conditions it assembles into an active decameric structure, resulting in a substantial increase in enzymatic activity [20,28]. As shown in Fig. 3A, our results are consistent with these findings, indicating that RDC undergoes a pH-dependent transition into a catalytically active higher-order assembly.

In contrast to RDC, DAO required a higher enzyme concentration of 4 mg/ml to ascertain measurable activity due to its slower reaction rates, whereas RDC was tested at 0.1 mg/ml. At a lower substrate concentration of 0.1 mM agmatine, DAO displayed minimal difference in activity between the 2 pH levels, suggesting a relatively stable activity profile at lower substrate availability. However, at a higher concentration of 1 mM agmatine, DAO exhibited a substantial increase in activity, approximately 2.4-fold higher at pH 6.4 compared to pH 7.4. This pronounced pH dependency at higher substrate concentrations can be attributed to the optimal pH range of DAO, which is between 6.4 and 7.0 [37]. The enhanced activity at pH 6.4 suggests that DAO may preferentially function in slightly acidic conditions, which could be particularly relevant in certain physiological environments where local acidity varies. Taken together, both RDC and DAO exhibit enhanced catalytic activity under acidic conditions, supporting their potential for tumor-selective activation.

Before assessing the cascade reaction, we first examined whether encapsulation affected the intrinsic catalytic activities of each enzyme. RDC and DAO were individually loaded into NCs, and their activities were compared with those of the free enzymes at pH 6.4. As shown in Fig. S6, no meaningful differences were observed between free and NC-encapsulated enzymes, indicating that the nanocarrier does not impair substrate accessibility or catalytic turnover. This confirms that any enhancement observed in the subsequent cascade experiments can be attributed primarily to spatial proximity and local confinement effects rather than changes in intrinsic enzyme activity.

The cascade reactions of free and co-encapsulated RDC and DAO were evaluated under 2 pH conditions (Fig. 3C). The concentration of RDC was kept constant, whereas the concentration of DAO was varied to achieve RDC:DAO in mass ratios of 1:1, 1:2, and 1:5. This design was based on the faster reaction rate of RDC relative to DAO, which is expected to influence the overall cascade reaction rate. Across all conditions, higher enzymatic activity was observed at pH 6.4 compared to pH 7.4, attributable to the higher reaction rates of RDC under mildly acidic conditions. Notably, at pH 6.4, the encapsulated system exhibited the greatest enhancement in cascade reaction rate at a 1:1 mass ratio, showing an approximately 5.1-fold increase, compared with 1.5-fold and 1.1-fold increases at 1:2 and 1:5 ratios, respectively. These results indicate that the close spatial proximity between RDC and DAO within NC enhances cascade efficiency. This enhancement is more pronounced at lower enzyme ratios, where intermolecular distances are expected to be greater in free solution, further supporting the nanoreactor effect.

In addition, the time-dependent retention of the cascade activity of NC-loaded RDC and DAO was evaluated at pH 6.4 to assess the stability of the nanoreactor system over time (Fig. S7). At each designated time point, after removing free enzymes released from the NC, the cascade reaction rate was subsequently measured using the enzymes retained within the NC. As a result, more than 50% of the initial cascade activity remained after 3 d compared to that at day 0. These findings demonstrate that the RDC/DAO@NC system preserves more than half of its catalytic activity after incubation for 3 d at 37 °C and pH 6.4, supporting the sustained functionality and stability of the nanoreactor system in the TME. Together with the unchanged intrinsic activities of individually encapsulated RDC and DAO (Fig. S6), these results suggest that the nanoreactor effect arises mainly from enzyme proximity within the NC, while retention of the enzyme pair contributes to sustained cascade activity over time.

### Enhanced anticancer effect in vitro through the cascade reaction between RDC and DAO in NC

To properly interpret the anticancer effects mediated by arginine deprivation, the argininosuccinate synthase 1 (ASS1) expression profile of SCC7 cells was first examined, as ASS1 expression in this cell line has not been previously characterized (Fig. S8). ASS1 mRNA levels were quantified by reverse transcription–quantitative polymerase chain reaction with Raw264.7 cells included as a reference ASS1-positive group. Notably, ASS1 transcription in Raw264.7 cells has been reported to be induced upon lipopolysaccharide (LPS) stimulation via signal transducer and activator of transcription 1-mediated signaling [38], and thus, LPS-treated Raw264.7 cells were used as a positive control. Consistent with this established mechanism, LPS-treated Raw264.7 cells exhibited a marked increase in ASS1 expression compared with untreated Raw264.7 cells. In contrast, SCC7 cells showed substantially lower ASS1 expression than even untreated Raw264.7 cells. These results confirm that the SCC7 cell line exhibits an ASS1-low expression phenotype, indicating an increased dependence on extracellular arginine, thereby providing a suitable model for evaluating arginine-deprivation-based therapy [24,39].

**Fig. 4.**
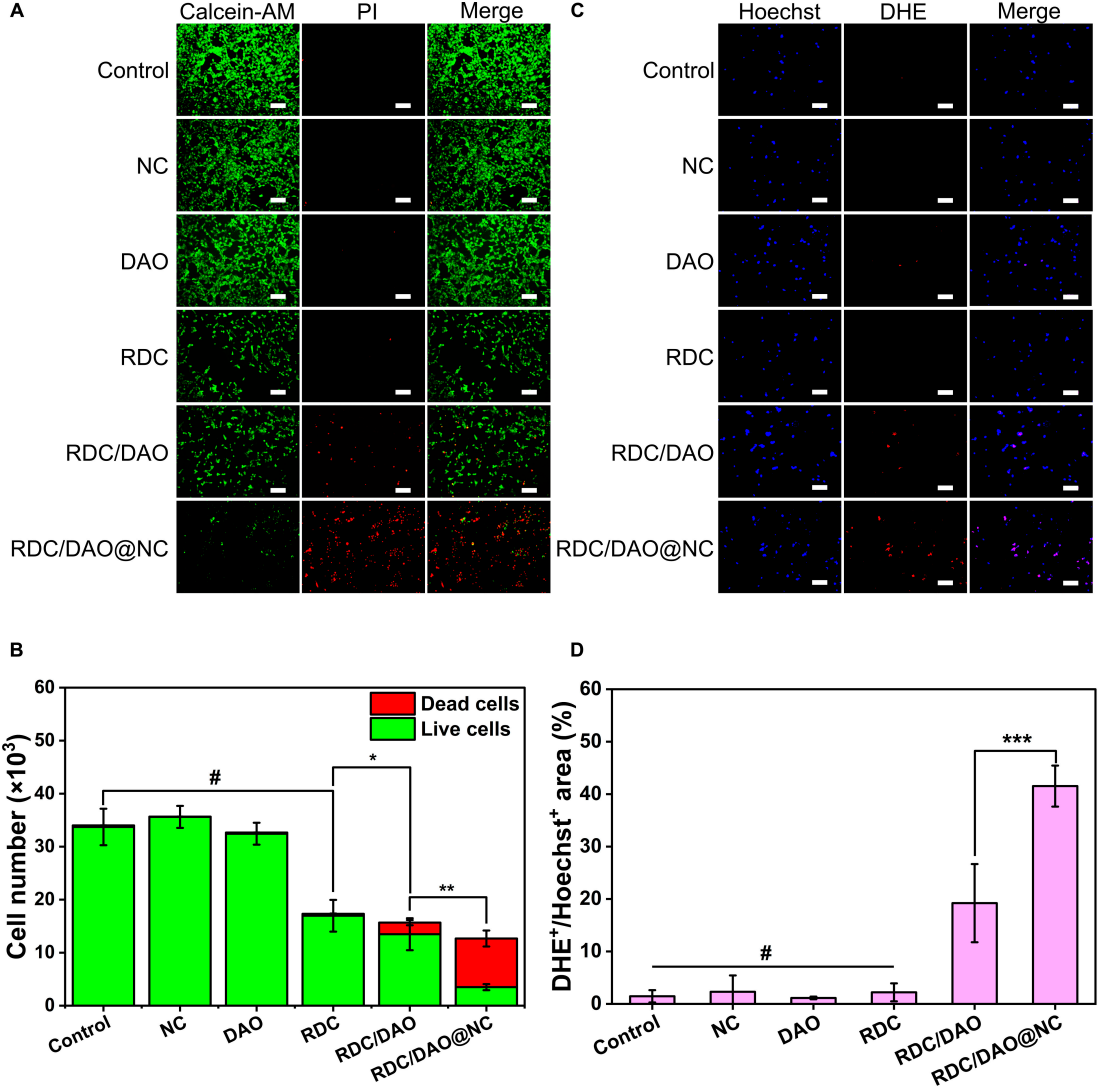
In vitro antitumor effects of RDC/DAO@NC on mouse squamous cell carcinoma 7 (SCC7) cells. (A) Fluorescence microscopy images of Live/Dead staining using calcein acetoxymethyl (Calcein-AM, green) and propidium iodide (PI, red) for 3-d incubation at pH 6.4 with 0.25 mg/ml of NC, 0.1 mg/ml of RDC, and 0.1 mg/ml of DAO. Scale bar = 100 μm. (B) Quantification of live and dead cells based on hemocytometer counts and Live/Dead staining areas. Statistical analysis on the dead cell population (*n* = 4). (C) Dihydroethidium (DHE)-staining images of SCC7 cells treated with 2.5 mg/ml of NC, 1 mg/ml of RDC, and 1 mg/ml of DAO for 1 d at pH 6.4. Scale bar = 100 μm. (D) Comparison of DHE-staining intensity (intracellular reactive oxygen species [ROS] level) normalized to Hoechst staining (total cell count) for different treatments (*n* = 5). Statistical significance: ^#^*P* > 0.05, *P < 0.05, **P < 0.01, and ***P < 0.001.

The enhanced anticancer effects of RDC and DAO mediated by their NC-facilitated cascade reaction were further evaluated. The SCC7 cells were treated with NC, RDC, DAO, RDC/DAO, or RDC/DAO@NC at pH 6.4 for 3 d. Following the treatment, the anticancer efficacy was assessed using a Live/Dead assay (Fig. 4A and B). The number of live and dead cells was quantified, and statistical analysis was performed on the dead cell populations. As expected, treatment with NC itself or DAO alone showed no effects on the cells in terms of cytotoxicity or proliferation. In the case of RDC, the number of dead cells was negligible (~2% of total cells); however, the total number of cells was substantially lower (~50%) compared to the nontreated group, indicating a pronounced reduction in cell proliferation. This substantial reduction in cell proliferation validates that the arginine starvation strategy was effective even in vitro.

When DAO was co-treated with RDC, the total number of cells was similar to the case of RDC alone, but dead cells increased more than 5-fold (from 0.4 × 10^3^ ± 0.1 × 10^3^ in the RDC group to 2.2 × 10^3^ ± 0.5 × 10^3^ in the RDC/DAO group). This indicates that the cascade reaction between RDC and DAO, producing H_2_O_2_, dramatically increased the cytotoxicity. When RDC and DAO were encapsulated in the NC and treated, the number of dead cells further increased over 4-fold (9.2 × 10^3^ ± 1.5 × 10^3^), compared to the RDC/DAO group. This clearly demonstrates that the NC could effectively amplify the cascade reaction, enhancing the conversion of arginine into H_2_O_2_. These results are consistent with the enzyme cascade kinetics described in the preceding section (Fig. 3).

To confirm that the cytotoxicity induced by RDC and DAO was attributable to H_2_O_2_, DHE staining was performed (Fig. 4C and D). No oxidative stress was detected in cells treated with NC, RDC, or DAO individually. In contrast, treatment with both RDC and DAO together resulted in clear oxidative stress within the cells. Notably, when RDC and DAO were encapsulated within the NC, oxidative-stress levels were markedly elevated compared to the non-encapsulated group. These findings align with the Live/Dead assay results, providing strong evidence that the observed cell death was mediated by H_2_O_2_ generated through the enzymatic cascade reaction between RDC and DAO and that the NC effectively enhanced this cascade process.

To further determine whether the oxidative stress generated by the RDC/DAO cascade reaction led to apoptotic cell death, Annexin V/PI staining was additionally performed (Fig. S9). No detectable Annexin V or PI signals were observed in cells treated with NC, RDC, or DAO alone. In contrast, co-treatment with RDC and DAO resulted in increased Annexin V- and PI-positive signals, indicating the induction of apoptosis. Notably, cells treated with RDC/DAO@NC exhibited a pronounced increase in both Annexin V and PI signals compared with the non-loaded RDC/DAO group. These results confirm that H_2_O_2_ generated through the NC-enhanced RDC/DAO cascade reaction induces cytotoxicity via apoptosis. Collectively, these findings suggest that the RDC/DAO@NC system enables an additional antitumor pathway beyond conventional arginine depletion therapy.

Previous studies have shown that RDC treatment in vitro inhibits tumor cell growth without inducing cell death, findings that align with our results (Fig. 4A and B) [26,28]. In this study, we enhanced the therapeutic efficacy of RDC by integrating DAO, achieving both growth inhibition and induction of cell death via H_2_O_2_ production. The NC effectively addressed a critical limitation of RDC by maximizing the cascade reaction, thereby substantially amplifying H_2_O_2_ production while simultaneously reducing the accumulation of intermediate metabolites such as agmatine. The markedly enhanced cascade efficiency observed at the cellular level highlights the NC’s exceptional functionality as a nanoreactor for both enzymes, emphasizing its potential as a transformative platform for cancer therapy.

The therapeutic effect of the present RDC/DAO system does not rely on intracellular delivery. Because SCC7 cells exhibit an ASS1-low phenotype and depend on exogenous arginine, depletion of L-arginine in the tumor extracellular milieu is expected to be sufficient to induce metabolic stress. In addition, H_2_O_2_ generated by the RDC/DAO cascade may exert cytotoxicity whether produced extracellularly or following cellular internalization. Therefore, efficient delivery and retention of the enzyme pair within tumor tissue is considered more critical than exclusive intracellular localization.

To further examine whether the RDC/DAO@NC system is limited by conventional arginine-deprivation therapy, which is typically effective only in ASS1-low expression cells, additional validation experiments were performed (Fig. S10). For this purpose, MCF7 cells, which are well known to express ASS1, were used as an ASS1-positive tumor model [40]. To first assess cellular sensitivity to arginine depletion, the metabolic activity of MCF7 and SCC7 cells was compared following treatment with RDC alone. In contrast to SCC7 cells, which exhibited a marked reduction in metabolic activity upon RDC treatment, MCF7 cells maintained high metabolic activity even at increasing RDC concentrations (Fig. S10A). These results further corroborate that SCC7 cells represent an ASS1-low expression cell line, consistent with the reverse transcription–quantitative polymerase chain reaction results shown in Fig. S8 and previous reports on ASS1 expression profiles in MCF7 cells [40]. Based on this result, MCF7 cells were subsequently treated with NC, RDC, DAO, RDC/DAO, and RDC/DAO@NC at pH 6.4 for 3 d to evaluate antitumor effects. There was no cytotoxicity of NC, DAO, and RDC alone in MCF7 cells. However, co-treatment with RDC and DAO showed measurable cytotoxicity, which was further enhanced when the enzymes were loaded in the NC (Fig. S10B). These results indicate that the RDC/DAO@NC system can partially circumvent the limitations of conventional arginine-deprivation therapy by introducing an additional ROS-mediated cytotoxic mechanism. Nevertheless, due to the intrinsic characteristics of this therapeutic strategy, the anticancer efficacy remains more pronounced in ASS1-low expression cells, while the effect in ASS1-expressing cells is relatively limited. Despite this limitation, the ability to extend therapeutic activity beyond arginine auxotrophy represents a meaningful advancement over conventional approaches.

In summary, while RDC suppressed cell proliferation without inducing apoptosis, substantial cell death was observed only when RDC operated in a cascade reaction with DAO, confirming that oxidative stress induced by H_2_O_2_ production is the primary driver of cytotoxicity. Importantly, this enzymatic cascade was selectively amplified under a mildly acidic environment (pH 6.4) when both enzymes were co-encapsulated within the NC, leading to a markedly enhanced anticancer effect. This pH-dependent amplification highlights the role of the NC as a nanoreactor that promotes efficient enzymatic cascade reactions in the TME. Together, these results suggest that the RDC/DAO@NC system may offer a complementary strategy to partially overcome the limitations of conventional arginine depletion therapy by integrating ROS-mediated cytotoxicity.

### Enhanced tumor-targeted delivery of RDC and DAO through NC

To analyze and demonstrate the tumor-targeted delivery of RDC and DAO through the NC, Alexa 680-conjugated RDC was utilized. As shown in Fig. 2B, both RDC and DAO were successfully loaded into the NC with ~80% efficiency. The release experiment of RDC and DAO (Fig. 2C and D) showed that both RDC and DAO were released more slowly upon the NC loading compared to free enzymes, so the NC could provide sustained release of RDC and DAO. Between them, RDC exhibited a faster release rate than DAO, indicating that DAO remains in the NC for a longer duration. Therefore, detection of RDC at the tumor upon NC-mediated delivery reasonably implies the co-delivery and retention of DAO within the TME.

**Fig. 5.**
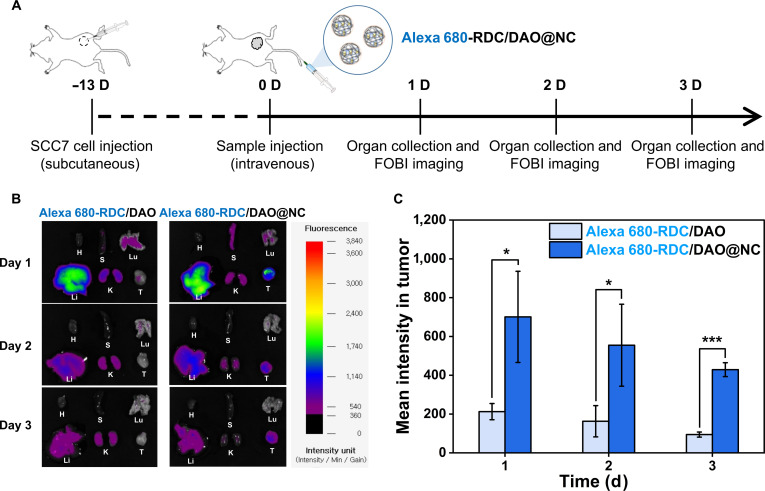
In vivo tumor targeting and biodistribution of RDC/DAO@NC. (A) Experimental timeline for biodistribution analysis following intravenous injection of Alexa 680-RDC/DAO or Alexa 680-RDC/DAO@NC into SCC7 tumor-bearing mice. (B) Representative ex vivo fluorescence images of major organs and tumor tissues collected on days 1, 2, and 3 (H: heart, S: spleen, Lu: lung, Li: liver, K: kidney, and T: tumor). (C) Quantification of mean fluorescence intensity in tumor tissues (*n* = 3). Statistical significance: *P < 0.05, ***P < 0.001.

To assess both the tumor-targeting ability and cargo-delivery performance of the NC, Alexa 680-conjugated RDC was co-loaded with DAO into the NC and intravenously injected into tumor-bearing mice. As a comparison group, Alexa 680-RDC and DAO were administered without NC loading. Fluorescence signals in major organs and tumors were analyzed on days 1, 2, and 3 after injection (Fig. 5).

Time-course biodistribution analysis revealed a substantial difference in tumor accumulation between the 2 groups. Alexa 680-RDC/DAO administered without NC showed minimal tumor fluorescence at all measured time points. In contrast, Alexa 680-RDC/DAO@NC exhibited markedly enhanced tumor accumulation 3.30-fold on day 1, 3.4-fold on day 2, and 4.6-fold on day 3 compared to the non-encapsulated group. This increasing trend over time suggests prolonged retention of the NC within the TME. These findings demonstrate the superior tumor-targeting efficiency of the NC and indicate that NC-mediated delivery enables prolonged tumor retention of the cargo for at least 3 d.

The present system was not designed to rely primarily on strong ligand-mediated active targeting. Instead, a chitosan-conjugated Pluronic-based NC platform was selected because it has been previously validated for tumor delivery and provides sufficient tumor accumulation for enzyme therapy [30,41,42]. Chitosan is not a classical receptor-specific targeting ligand, but chitosan-containing nanocarriers have been widely used to improve tumor-associated delivery and retention [43]. In this study, a strong active-targeting ligand was not employed because the primary aims were to enable co-delivery of RDC and DAO while preserving their enzymatic activity and to emphasize the tumor-selective activation of RDC under mildly acidic conditions and the cascade reaction with DAO.

Outside the tumor, fluorescence signals were predominantly detected in the liver and kidney, gradually decreasing over time (Figs. S11 and S12). This pattern is consistent with the typical clearance routes of nanoscale formulations, in which particles undergo hepatic processing and renal elimination [44]. The progressive reduction in off-target organ accumulation supports the safe clearance profile of the NC while maintaining its prolonged presence in the TME. Additionally, pharmacokinetic analysis (Fig. S13) demonstrated that NC encapsulation substantially prolonged the systemic circulation of loaded enzymes, thereby supporting the improved in vivo availability and delivery efficiency mediated by the NC.

Collectively, these results demonstrate that the NC effectively facilitated the tumor-specific accumulation of RDC, mitigating its inherently short half-life, a major limitation of RDC-based therapy. Furthermore, consistent with our previous report [30], the NC exhibited good tumor-targeting efficiency, enabling the successful co-delivery of RDC and DAO to the tumor site. These findings highlight the dual role of the NC as both a delivery vehicle that improves in vivo pharmacokinetics and a nanoreactor that enhances the enzymatic cascade reaction.

### Superior anticancer effects of RDC/DAO@NC in vivo via targeted tumor delivery and amplified enzyme cascade reaction

**Fig. 6.**
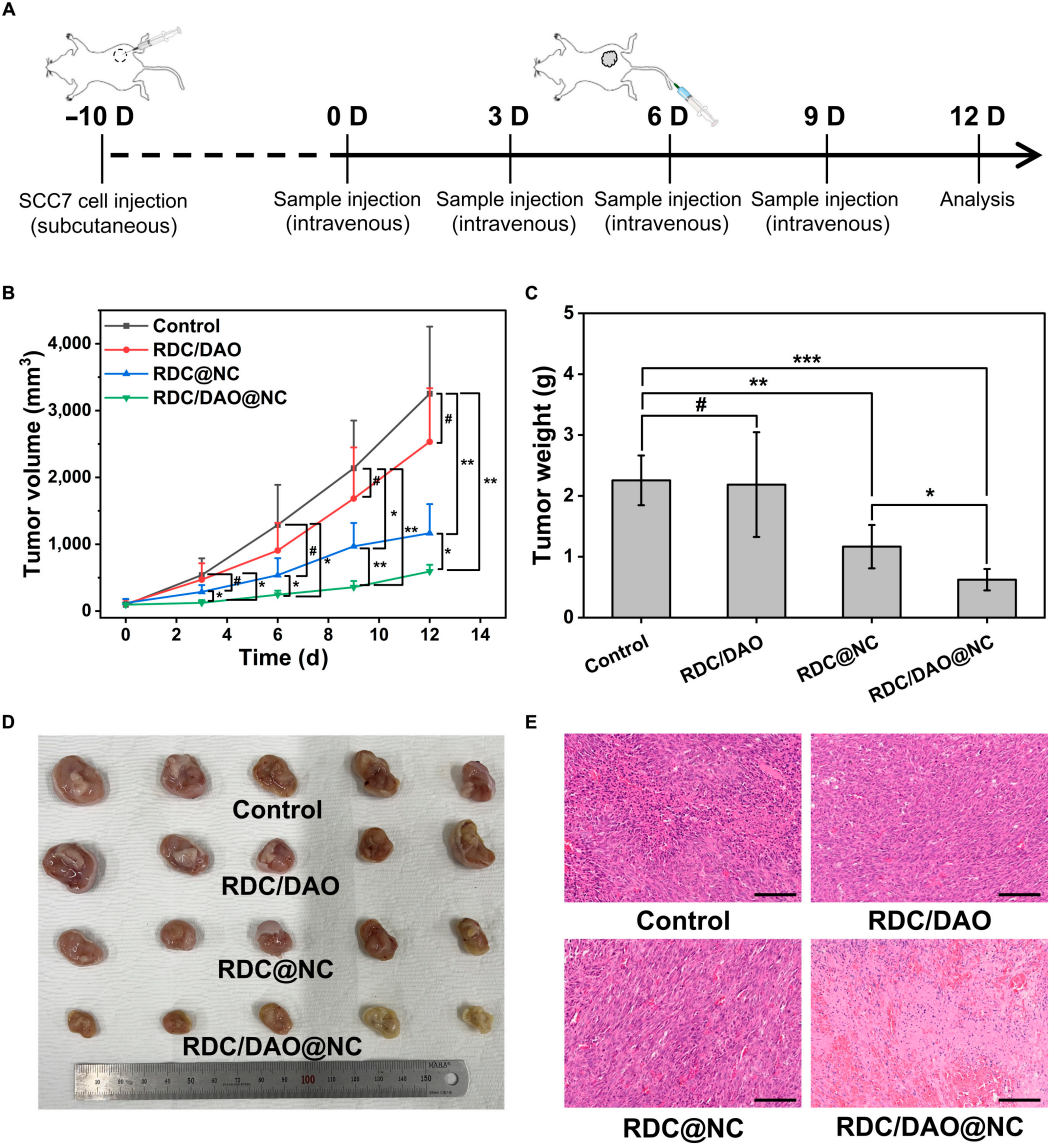
In vivo antitumor effect of RDC/DAO@NC. (A) Experimental timeline for tumor model formation and sample injection. (B) Tumor growth curves of SCC7 tumor-bearing mice treated with phosphate-buffered saline (control), RDC/DAO, RDC@NC, or RDC/DAO@NC (40 mg/kg RDC, 40 mg/kg DAO, and 100 mg/kg NC) (*n* = 5). Tumor volumes were measured every 3 d. (C) Tumor weights of mice harvested 12 d after initial sample injection (*n* = 5). (D) Images of tumors excised from each treatment group. (E) Representative hematoxylin-and-eosin-stained images of tumor sections from each group, highlighting histological changes. Scale bars = 100 μm. Statistical significance: ^#^*P* > 0.05, *P < 0.05, **P < 0.01, and ***P < 0.001.

**Fig. 7.**
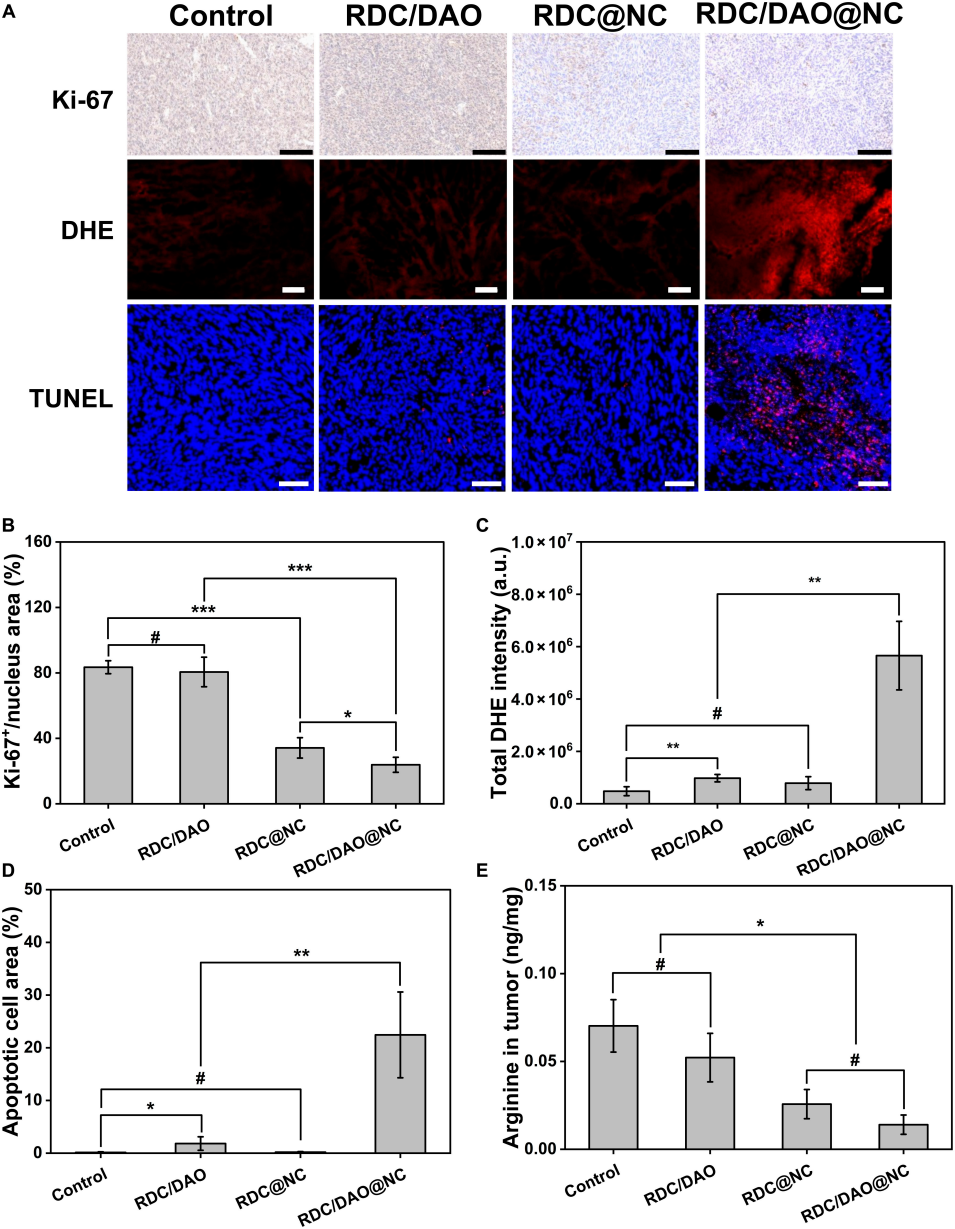
Ex vivo tumor analysis. (A) Representative Ki-67-immunostaining, DHE-staining, and terminal deoxynucleotidyl transferase dUTP nick end labeling (TUNEL)-staining images of tumor sections from each treatment group. Ki-67 staining (brown) with hematoxylin (nuclei, blue) counterstaining to evaluate tumor cell proliferation. DHE staining (red) to visualize intracellular ROS levels. TUNEL staining (red) with Hoechst (nuclei, blue) counterstaining to indicate TUNEL-positive apoptotic cells. Scale bars = 100 μm. (B) Quantification of Ki-67-positive area normalized to total nuclei (*n* = 5). (C) Quantification of total DHE fluorescence intensity in tumor tissues (*n* = 5). (D) Quantification of TUNEL-positive apoptotic area (*n* = 5). (E) Intratumoral arginine levels measured by competitive enzyme-linked immunosorbent assay (*n* = 3). Statistical significance: ^#^*P* > 0.05, *P < 0.05, **P < 0.01, and ***P < 0.001.

Given that NC was shown to amplify the cascade reaction of RDC and DAO while facilitating efficient tumor-targeted delivery, and that more than 50% of the cascade catalytic activity was retained for up to 3 d under tumor-relevant conditions, its antitumor therapeutic efficacy was evaluated (Figs. 6 and 7). After tumor formation, the samples were administered intravenously every 3 d for a total of 4 injections (Fig. 6A). When RDC and DAO were administered without using NC encapsulation (RDC/DAO), no meaningful anticancer effect compared to the control group in terms of tumor size was observed. This outcome can be attributed to the inherently short in vivo half-life of RDC, which leads to rapid systemic clearance when the enzyme is administered without NC. This interpretation is well supported by the biodistribution and pharmacokinetics data shown in Fig. 5 and Fig. S13, respectively. As demonstrated in these figures, only a minimal amount of free RDC accumulated in the tumor sites, while plasma levels declined rapidly over time, indicating that direct administration of bare enzymes is ineffective for tumor-targeted delivery.

In contrast, when RDC alone was encapsulated within the NC (RDC@NC) for delivery, a noticeable suppression of tumor growth was observed. This result, consistent with the cellular-level findings (Fig. 4), validates the efficacy of arginine deprivation in inhibiting tumor progression in an in vivo model. However, both our data (Fig. 4) and previous in vitro studies [26] showed that while RDC alone could impede the proliferation of tumor cells, it was insufficient to induce tumor cell death. To further enhance therapeutic efficacy, DAO was co-loaded with RDC into the NC (RDC/DAO@NC) and administered. This combination resulted in a substantial reduction in both tumor size and weight compared to the RDC@NC group (Fig. 6B to D and Fig. S14). Furthermore, histological analysis of tumor tissues via H&E staining (Fig. 6E) revealed a pronounced decrease in tumor compactness exclusively in the RDC/DAO@NC group. This finding underscores the enhanced therapeutic efficacy of a dual-enzyme system facilitated by NC encapsulation, highlighting its potential as a highly effective anticancer strategy. While previous studies have reported the anticancer effects of RDC at the cellular level, its therapeutic efficacy in animal models has remained unexplored [26,28]. This study holds important value as the first report to demonstrate the anticancer potential of RDC in an in vivo model, leveraging NC to enhance its stability and tumor-targeted delivery, thereby overcoming its inherent limitations.

To verify the individual contributions of RDC and DAO to the anticancer effect, Ki-67 immunostaining, quantification of intratumoral arginine level, DHE staining, TUNEL assay, and Western blot analysis were performed (Fig. 7 and Fig. S15). The evaluation focused on whether arginine depletion mediated by RDC, along with the ROS-generating cascade reaction between RDC and DAO, could suppress tumor cell proliferation and induce tumor cell death in vivo.

Ki-67 is a well-established protein marker associated with cellular proliferation, as it is expressed in all active phases of the cell cycle (G1, S, G2, and M), except for the quiescent phase (G0) [45]. It serves as a critical biomarker for evaluating tumor proliferative activity and is widely used in cancer diagnostics and prognostic assessment. As shown in Fig. 7A and B, tumors treated with free RDC and DAO without NC encapsulation (RDC/DAO) showed no reduction in Ki-67-positive cells, indicating that bare enzyme administration was ineffective. In contrast, when RDC was effectively delivered to the tumor site via NC (RDC@NC), a substantial decrease in Ki-67 expression was observed, confirming the efficacy of NC-mediated arginine deprivation in effectively inhibiting tumor cell proliferation in vivo. Notably, the co-delivery of RDC and DAO via NC (RDC/DAO@NC) led to an even more pronounced reduction in Ki-67 expression, consistent with the enhanced antitumor efficacy observed in Fig. 6.

To determine whether the observed suppression of tumor cell proliferation originated from RDC-mediated metabolic starvation, intratumoral arginine levels were quantified using an enzyme-linked immunosorbent assay and normalized to tumor mass (ng/mg). As shown in Fig. 7E, control tumors contained 0.070 ± 0.015 ng/mg of arginine. Free RDC/DAO caused only a modest reduction (0.052 ± 0.014 ng/mg; ~25% decrease). In contrast, RDC@NC substantially reduced arginine levels to 0.026 ± 0.008 ng/mg (~63% reduction). Notably, RDC/DAO@NC resulted in the most profound depletion (0.014 ± 0.006 ng/mg; ~80% decrease). These results clearly demonstrate that nanocarrier-mediated enzyme delivery markedly enhances in vivo arginine depletion. Collectively, these findings support that the tumor growth inhibition observed in the RDC@NC and RDC/DAO@NC groups primarily originates from RDC-driven metabolic starvation in the acidic TME. Taken together with the successful tumor delivery of the enzyme via NC (Fig. 5), these results indicate that the NC enables delivery of the enzymatic payload while retaining its functional activity, rather than acting solely as a passive carrier.

To determine whether RDC activation, as evidenced by decreased intratumoral arginine levels, led to a subsequent cascade reaction and resulted in ROS production in tumor tissues, DHE staining was performed on tumor sections (Fig. 7A and C). The RDC/DAO group exhibited a slightly increased DHE signal compared with the control group, and the RDC@NC group, in which successful tumor delivery of RDC was confirmed, also did not show a noticeable increase in DHE signal. This observation further supports that RDC alone primarily contributes to arginine starvation rather than ROS generation. In contrast, the RDC/DAO@NC group showed a markedly elevated DHE signal, indicating substantial ROS generation in the tumor tissue. These results indicate that activated RDC successfully triggered the cascade reaction with DAO and that DAO was also delivered to the tumor site in a functionally preserved state via the NC.

To further assess whether the ROS generated at the tumor site, as evidenced by DHE staining, resulted in actual apoptosis in the tumor tissue, a TUNEL staining was performed on tumor sections (Fig. 7A and D). In the RDC/DAO group, only minimal apoptosis was detected, consistent with previous findings indicating that non-encapsulated enzymes exhibited poor tumor localization and failed to induce a cascade reaction effectively. Similarly, in the RDC@NC group, although tumor proliferation was substantially suppressed, apoptosis remained negligible. As shown in Fig. 4, RDC treatment effectively reduced the total tumor cell population but did not induce cell death, suggesting that arginine deprivation alone is insufficient to trigger apoptosis in this system. This observation is consistent with prior studies demonstrating that arginine-deiminase-mediated arginine depletion also fails to induce direct tumor cell death [46]. In contrast, the RDC/DAO@NC group exhibited a substantial increase in apoptotic tumor cells, demonstrating that the cascade reaction with DAO was essential for inducing cell death, which could not be achieved by RDC alone. This result suggests that the RDC and DAO cascade reaction led to sufficient intratumoral H_2_O_2_ generation, ultimately driving oxidative-stress-induced apoptosis.

Following the apoptosis observed by TUNEL staining, the apoptotic signaling pathway was further investigated by analyzing caspase-3 activation in tumor tissues. Proteins were isolated from tumor samples, and the expression levels of caspase-3 and cleaved caspase-3 were examined by Western blot analysis (Fig. S15). Inactive procaspase-3 is cleaved by upstream caspases into its active form, and cleaved caspase-3 subsequently executes apoptosis by targeting and dismantling cellular components [47]. In tumors treated with free RDC/DAO, only a very weak signal of cleaved caspase-3 was detected. In contrast, the RDC/DAO@NC group exhibited a markedly elevated level of cleaved caspase-3. These results indicate that the RDC/DAO@NC system effectively induces apoptosis in tumor tissues through activation of the caspase-3-dependent pathway. This trend is consistent with the increased apoptotic cell population observed in the TUNEL-staining results, further confirming that the enhanced enzymatic cascade reaction mediated by NC leads to actual apoptosis in vivo.

Collectively, these results support 2 distinct and complementary antitumor mechanisms of RDC/DAO@NC. First, the acidic TME activates the encapsulated RDC, leading to intratumoral arginine depletion and suppression of tumor growth through metabolic starvation. Second, the agmatine generated by RDC is subsequently oxidized by DAO to produce H_2_O_2_, and the resulting ROS accumulation induces apoptosis in tumor tissues, thereby further enhancing the overall antitumor efficacy.

### In vivo biocompatibility of RDC/DAO@NC

To evaluate the biocompatibility of RDC/DAO@NC, body weight changes were monitored following sample administration. Additionally, 3 d after the final injection, histological analysis of major organs (heart, liver, lungs, spleen, and kidneys) was conducted alongside serum biochemical analysis to evaluate kidney function (BUN and creatinine) and liver function (AST and ALT) (Fig. S16). Throughout the 4 intravenous administrations over 3 d, no meaningful differences in body weight were observed in all groups (Fig. S16A). Serum BUN and creatinine levels remained within the normal range in all groups, and AST and ALT levels showed no meaningful differences, indicating the absence of systemic toxicity (Fig. S16B to E). H&E staining of major organs revealed no detectable histopathological abnormalities, further confirming the absence of organ toxicity (Fig. S16F). Collectively, these findings demonstrate that RDC, DAO, and NC did not produce detectable toxicity at the tested doses.

The NC employed in this study enabled efficient tumor-targeted delivery of enzymatic payloads while preserving their functional activity rather than merely serving as a passive transport carrier. Furthermore, the incorporation of DAO introduced an additional apoptosis-inducing mechanism in metabolically starved tumors through H_2_O_2_ generation, thereby providing a complementary antitumor pathway beyond arginine deprivation alone. The dual functionality of the NC, as a tumor-targeting delivery system and a nanoreactor that amplifies the enzymatic cascade, underpins this strategy and contributes to superior anticancer efficacy compared with conventional arginine-deprivation therapy. In this cascade system, agmatine is generated as an intermediate from arginine via RDC and is subsequently oxidized by DAO to produce hydrogen peroxide (H_2_O_2_), thereby linking arginine depletion to localized ROS generation. While agmatine has been reported to influence cellular processes such as polyamine metabolism and apoptosis [48,49], its accumulation is expected to be limited in this system due to rapid enzymatic conversion by DAO within the localized cascade environment. Together, these findings suggest that the RDC/DAO@NC platform represents a rational strategy to extend the therapeutic applicability of arginine starvation therapy by integrating arginine depletion with ROS-mediated apoptosis. Although RDC/DAO@NC showed no detectable short-term systemic toxicity under the present dosing schedule, long-term biocompatibility will require dedicated investigation in future translational work.

Enzyme-based metabolic therapies have been clinically established, as exemplified by L-asparaginase, which is widely used for the treatment of acute lymphoblastic leukemia; however, systemic exposure of such enzymes is associated with various toxicities, including hypersensitivity, pancreatitis, and hepatotoxicity [50]. In addition, metabolic targeting strategies such as arginine deprivation have been widely explored for cancer therapy due to tumor dependence on extracellular arginine [27], yet these approaches generally lack intrinsic control over enzymatic activity and remain active under physiological conditions.

In contrast, our system introduces a tumor-responsive enzymatic cascade in which RDC is selectively activated under mildly acidic tumor conditions, thereby providing selectivity at the level of enzymatic activity itself. This pH-dependent activation, combined with cascade amplification via DAO and nanocarrier-mediated delivery, provides an additional layer of tumor selectivity and may contribute to improving the therapeutic window by minimizing systemic activity while enhancing localized therapeutic efficacy.

## Conclusion

This study demonstrates the first application of RDC as a tumor-responsive therapeutic enzyme by exploiting its pH-dependent activation under mildly acidic conditions. RDC-mediated arginine depletion effectively suppresses tumor cell proliferation; however, its therapeutic effect is primarily cytostatic when used alone.

To address this limitation, RDC was combined with DAO to establish an enzymatic cascade in which agmatine, generated by RDC, is further converted into H_2_O_2_. This cascade reaction links metabolic deprivation to localized ROS generation, resulting in a transition from growth inhibition to apoptosis-mediated tumor cell death. Importantly, the rapid conversion of agmatine by DAO is expected to limit its accumulation, while simultaneously amplifying cytotoxicity through H_2_O_2_ production.

Furthermore, co-encapsulation of RDC and DAO within the nanocarrier (RDC/DAO@NC) enabled efficient tumor accumulation, prolonged retention, and preservation of enzymatic activity in vivo. This system facilitates close spatial proximity between the 2 enzymes, promoting an effective cascade reaction within the TME. As a result, the combined strategy integrates pH-dependent enzyme activation, metabolic depletion, and ROS-mediated apoptosis, leading to enhanced antitumor efficacy compared to either enzyme alone.

Collectively, this work demonstrates that the integration of tumor-responsive enzyme activation with cascade amplification and nanocarrier-mediated delivery can overcome key limitations of conventional arginine-deprivation therapy. This strategy provides a rational framework for developing more effective enzyme-based cancer therapies by coupling metabolic targeting with localized cytotoxic amplification.

## Data Availability

Data will be made available upon request.
