# Supplementary material for: A Tumor-Responsive Enzymatic Cascade System Inducing pH-Activable Metabolic Starvation and H2O2-Induced Apoptosis
Source: Biomater Res. 2026 Jun 9;30:0380. doi: 10.34133/bmr.0380 (PMC13247311; doi:10.34133/bmr.0380)
Supplement: Supplementary 1 — Figs. S1 to S16 Table S1 [file bmr.0380.f1.docx]

**Supplementary materials**

A tumor-responsive enzymatic cascade system inducing pH-activable metabolic starvation and H_2_O_2_-induced apoptosis

Junyoung Jung^†^, Jae Hun Lee^†^, Seoungkyun Kim, Kiyoon Kwon, Giyoong Tae*, and Inchan Kwon*

**Materials**

Pluronic 68 (PF 68) was kindly donated by BASF Corp. (Seoul, Korea). Water-soluble chitosan (10 kDa, deacetylated degree = 85 %) was purchased from Amicogen (Seoul, Korea). Acryloyl chloride, glycidyl methacrylate (GMA), and Propidium Iodide (PI) were purchased from Sigma Aldrich (Saint Louis, MO, USA). Iragacure 2959 was purchased from Chiba Specialty Chemicals (Basel, Switzerland). Cellulose ester dialysis bag (MWCO 50 kDa) was purchased from Spectrum (Houston, TX, USA). Cy5.5-mono NHS ester was purchased from GE Healthcare (Pittsburgh, PA, USA). Trifluoroacetic acid (TFA; 99%) was sourced from Daejung Chemicals & Metals (Gyeonggi, South Korea). Nanosep® centrifugal filter (MWCO 300 kDa) was purchased from Pall Life Sciences (Ann Arbor, MI, USA). PD-10 desalting columns were sourced from GE Healthcare (Piscataway, NJ, USA). The Mighty Mix DNA Ligation Kit was purchased from TaKaRa Bio Inc. (Kusatsu, Japan). The AccuPrep® Nano-Plus Plasmid Mini Extraction Kit was purchased from Bioneer (Daejeon, South Korea). Ninitrilotriacetic acid (Ni-NTA) agarose beads and polypropylene columns were purchased from Qiagen (Hilden, Germany). DMEM, Fetal bovine serum (FBS), Antibiotic-Antimycotic (AA), TOP10 E. coli cells and isopropyl-β-D-thiogalactopyranoside (IPTG), Hoechst 33342, High-capacity cDNA reverse transcription kit, PowerUp^TM^ SYBR^TM^ Green Master Mix, Bolt^TM^ LDS Sample Buffer, Bolt^TM^ sample Reducing Agent, Bolt^TM^ Bis-Tris Plus Mini Gels, Bolt^TM^ MES SDS Running buffer, GAPDH GA1R, Goat anti-Rabbit IgG (H+L) HRP, iBlot™ 3 mini PVDF Transfer Stacks, and Rabbit anti-Mouse IgG (H+L) HRP were purchased from Thermo Fisher Scientific (Waltham, MA, USA). Tri-RNA Reagent was purchased from Favorgen (Ping Tung, Taiwan). Caspase-3 and cleaved caspase-3 antibody were purchased from Cell Signaling Technology (Danvers, MA, USA). A steel target plate and Protein Standard II were obtained from Bruker (Billerica, MA, USA). BamHI and SpeI enzymes were obtained from New England Biolabs (Ipswich, MA, USA). Hematoxylin and Eosin Y were purchased from BBC Biochemical (Mount Vernon, CA, USA). Yeast extract, tryptone, and agar were acquired from DB Biosciences (San Jose, CA, USA). Calcein-AM, Dihydroethidium (DHE), Alexa Fluor^TM^ 488-Annexin V, Alexa Fluor^TM^ 680 NHS Ester (Succinimidyl Ester), Ki-67 recombinant rabbit monoclonal antibody, Click-it^TM^ Plus TUNEL Assay kits, and Urea Nitrogen Colorimetric Detection kit were purchased from Invitrogen (Carlsbad, CA, USA). Rabbit-specific HRP/DAB (ABC) Detection IHC kit was purchased from Abcam (Cambridge, UK). A creatinine assay kit was purchased from BioAssay Systems (Hayward, CA, USA). GOT assay kit and GPT assay kit were purchased from Asanpharm (Seoul, Korea). L-Arginine Competitive EIA/ELISA Kit was purchased from LifeSpan BioSciences (CA, USA).

**S1. Preparation and characterization of chitosan-conjugated nanocarrier**

The Nanocarrier (NC) was prepared by photo-polymerizing diacrlyated Pluronic F68 (DA-PF68) and glycidyl methacrylated-chitosan (GMA-chitosan), following a previously reported protocol [30]. Briefly, GMA-chitosan (0.2 μmol) was dissolved in deionized water (DIW) and mixed with a solution of DA-PF68 (1.5 μmol). The resulting mixture was exposed to UV light (1.3 mW/cm^-2^) for 15 min in the presence of Irgacure 2959 (0.057 wt%), allowing incorporation of GMA-chitosan into the nanocarrier *via* the vinyl groups during the photo-polymerization of DA-PF68. Unreacted GMA-chitosan was removed through dialysis using a cellulose ester membrane (MWCO of 50 kDa).

The ^1^H NMR spectrum of NC in DMSO-D_6_ was obtained using a 600 MHz NMR spectrometer (Advance Neo 600, Bruker, MA, USA). ATR-FTIR analysis of NC was performed using a FTIR spectrometer (Vertex 70v, Bruker, MA, USA).


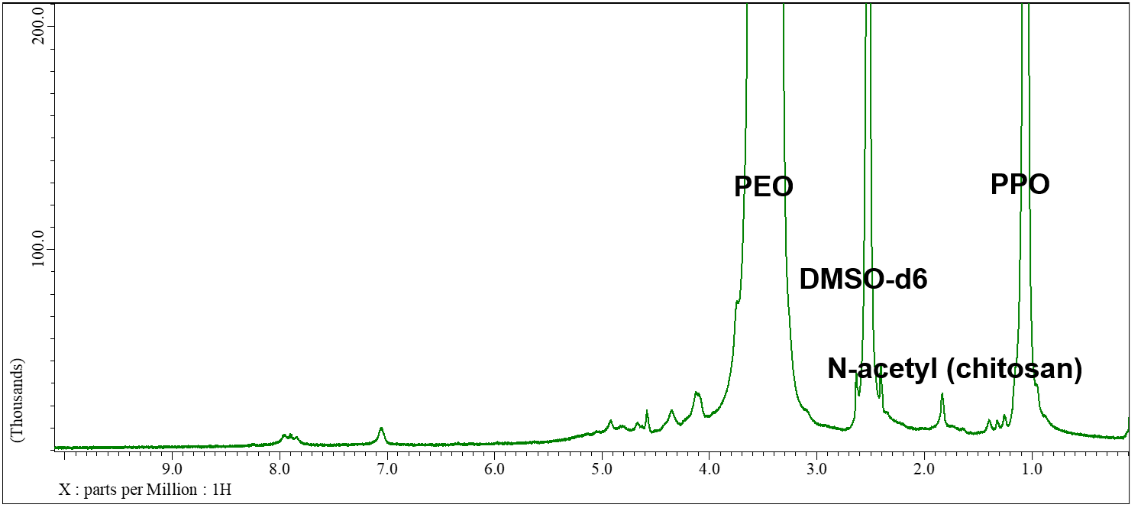


**Figure S1**. ^1^H NMR spectrum of NC in DMSO-d_6_. Peaks corresponding to the PEO protons (δ 3.4-3.6 ppm) and PPO protons (δ 0.9-1.0 ppm) of Pluronic, as well as the N-acetyl methyl group of chitosan (δ 1.9-2.0 ppm), are indicated.

**
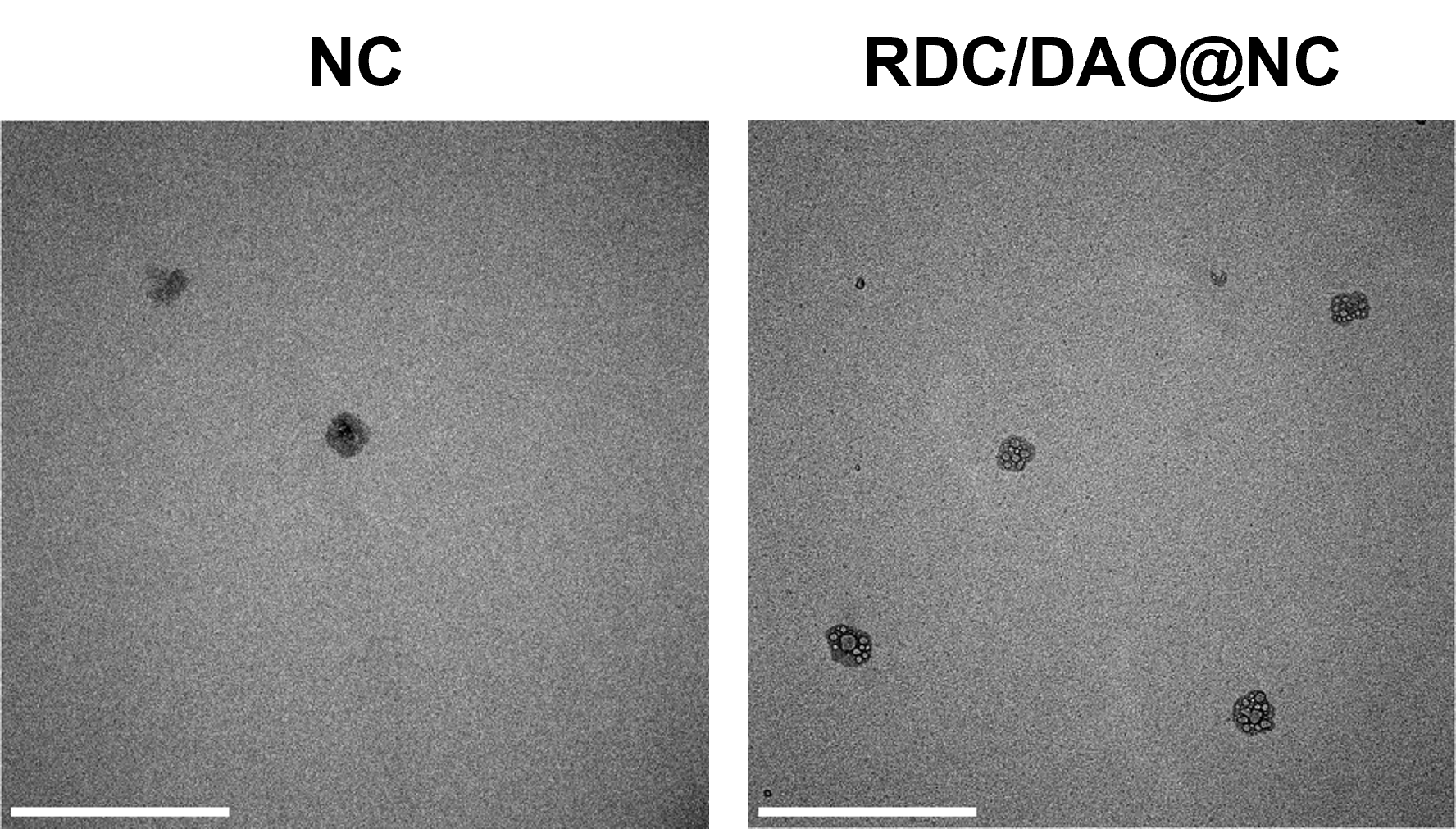
**

**Figure S2.** Transmission electron microscopy (TEM) images of the NC and RDC/DAO@NC. Scale bar = 500 nm**.**


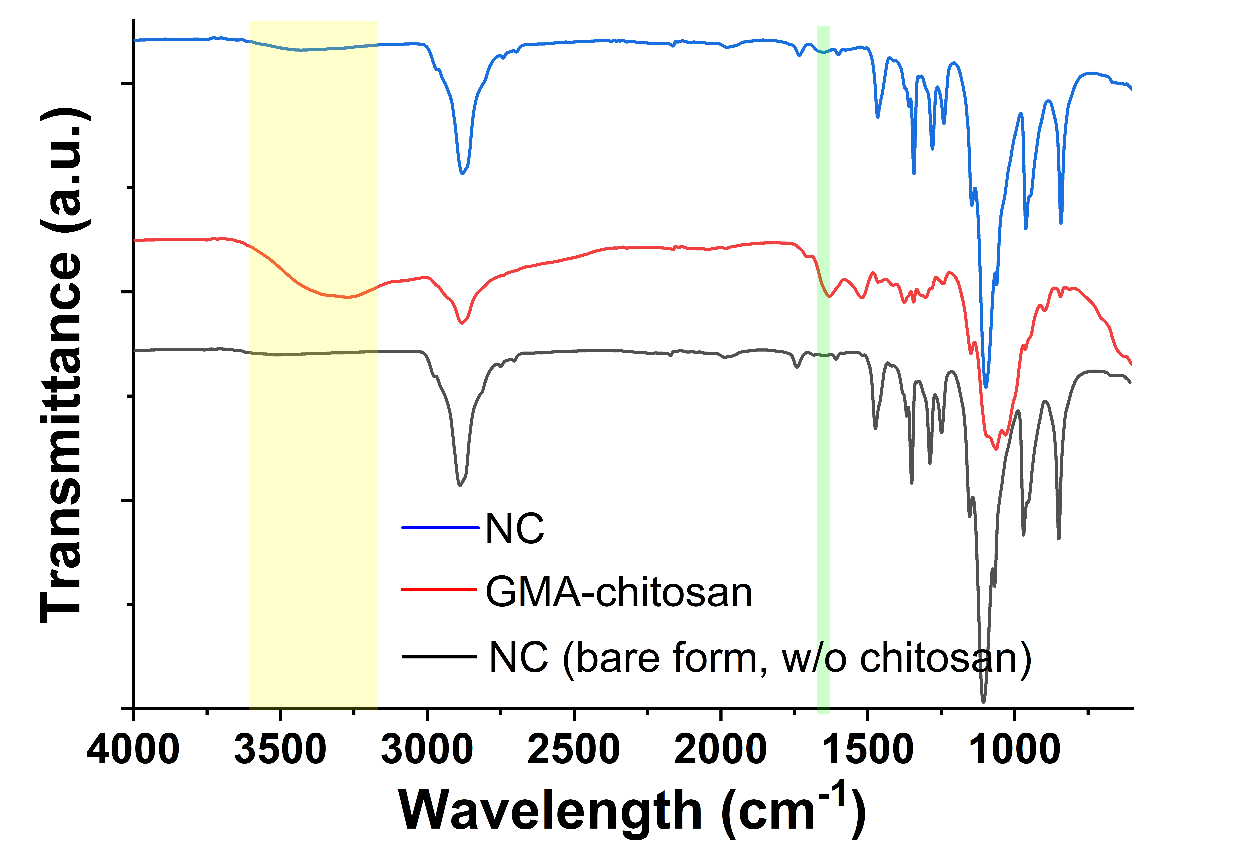


**Figure S3**. ATR-FTIR spectra of NC, Glycidyl Methacrylate-chitosan (GMA-chitosan), and NC (bare form, without chitosan). NC was prepared by photo-polymerization of diacrylated Pluronic F68 with GMA-chitosan, whereas the bare form was generated by photo-polymerizing diacrylated Pluronic F68 alone without GMA-chitosan. The yellow box highlights the N-H/O-H stretching region (3200-3600 cm^-1^) and the green box highlights the amide Ⅰ band (1650 cm^-1^).

**S2. Preparation of RDC**

**S2.1 Plasmid construction of RDC**

To generate a plasmid encoding arginine decarboxylase (RDC) from *Escherichia coli*, genomic DNA was extracted from the K12 strain using the AccuPrep® Nano-Plus Plasmid Mini Extraction Kit (Bioneer, Korea). The RDC gene, tagged with a hexa-histidine sequence for purification, was amplified by PCR using the C1000 Touch Thermal Cycler (Bio-Rad, USA). Amplification was performed using primers containing *BamH*I and *Spe*I restriction sites: forward 5’-ATCGGGATCCATGAAAGTATTAATTGTTGAAAGCGAG-3’ and reverse 5’-ATCGACTAGTTAATGGTGATGGTGATGGTGCGCTTTCACGCACATAAC-3’. The amplified product was digested with *BamH*I and *Spe*I enzymes (New England Biolabs, USA) and ligated into the pQE80 vector using the Mighty Mix DNA Ligation Kit (TaKaRa Bio Inc., Japan) to generate the pQE80-RDC plasmid. The ligation mixture was transformed into chemically competent TOP10 *E. coli* cells (Thermo Fisher Scientific, USA) and plated on LB agar containing 100 μg/mL ampicillin. Positive colonies were identified by colony PCR, and the successful ligation was confirmed by double digestion with *BamH*I and *Spe*I, followed by gel electrophoresis using the Gel Doc™ XR+ System (Bio-Rad, USA).

**S2.2 Expression and purification of RDC**

The pQE80-RDC plasmid was transformed into the TOP10 *E. coli* strain and plated on LB agar containing 100 µg/mL ampicillin, followed by overnight incubation at 37 ℃. A single colony was inoculated into 2.5 mL of LB medium with ampicillin (100 µg/mL) and cultured at 37 ℃ and 210 rpm in a shaking incubator overnight. The culture was scaled up by transferring 2 mL into 200 mL of 2xYT medium in a 1,000 mL flask, followed by incubation until OD at 600 nm (OD_600_) reached 0.4-0.5, measured using a microplate reader (BioTek, USA). A 1 mL sample was taken for SDS-PAGE analysis (Before Induction, BI). Protein expression was induced with 1 mM IPTG when OD_600_ reached 0.5-0.6. The culture was incubated at 18 ℃ for 24 h at 210 rpm. After induction, another sample was taken for SDS-PAGE analysis (After Induction, AI). Cells were harvested by centrifugation at 8,000 RCF for 10 min at 4 ℃.

For lysis, cell pellets were resuspended in lysis buffer (50 mM NaH_2_PO_4_, 300 mM NaCl, and 10 mM imidazole, pH 8.0) at 10 mL per gram of pellet. The suspension was treated with lysozyme (5 mg/mL), DNase I (5 µg/mL), and RNase A (10 µg/mL) and sonicated on ice using SONICS Ultrasonic Processor VCX500 (USA) (32% amplitude, 1-sec pulses, 2-sec rest, 15 min). After sonication, the lysate was centrifuged at 10,000 ×g for 25 min at 4 ℃.

The supernatant was incubated with Ni-NTA agarose (Bioneer, South Korea) at 4 ℃ for 1 h, with gentle rocking. The mixture was then loaded onto a polypropylene column (Qiagen, Germany). The column was washed with buffer (50 mM NaH_2_PO_4_, 300 mM NaCl, and 20 mM imidazole, pH 8.0). RDC was eluted with an elution buffer containing 250 mM imidazole. For buffer exchange, eluted proteins were processed using PD-10 Desalting Columns (GE Healthcare, USA) equilibrated with PBS.


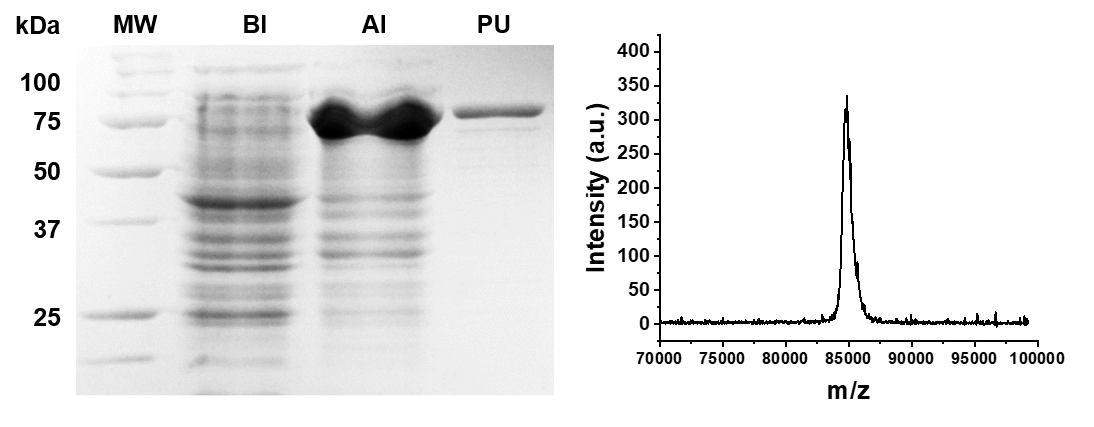


**Figure S4**. SDS-PAGE analysis of RDC expression and purification. Lane 1: Protein ladder (LD); Lane 2: Whole cell lysate before induction (BI); Lane 3: Whole cell lysate after induction (AI); Lane 7: Purified protein (PU).

**S3. Preparation and characterization of RDC/DAO@NC**

**S3.1 Preparation of RDC/DAO@NC**

The temperature-responsive volumetric changes of the NC were employed to encapsulate RDC and DAO, as previously reported for protein loading [31, 32]. Solutions of RDC and DAO were prepared in PBS (0.1 M, pH 7.4) at a concentration of 20 mg/mL. A 40 μL aliquot of each enzyme solution was added to 100 μL of NC solution (20 mg/mL in PBS) and incubated at 4 ℃ overnight to induce volume expansion of the NC. The mixture was then transferred to a 37 ℃ incubator for 15 min, promoting spontaneous enzyme encapsulation *via* NC size reduction. Unloaded RDC and DAO were separated by centrifugation at 8,000 rpm for 40 min using a Nanosep^®^ centrifugal filter (MWCO 300 kDa), resulting in the collection of RDC- and DAO-loaded NC (RDC/DAO@NC). Similarly, RDC alone was encapsulated into NC to obtain RDC@NC.

**S3.2 Characterization of RDC/DAO@NC**

The hydrodynamic size and surface charge of NC and RDC/DAO@NC were measured using an electrophoretic light scattering system (ELSZ-2000, Otsuka Electronics, Osaka, Japan) at 37 ℃. The morphologies of NC and RDC/DAO@NC were imaged by transmission electron microscopy (Talos F200X G2, Thermo Fisher, Waltham, MA, USA).

To assess the encapsulation efficiency of RDC and DAO, Alexa488 dye was conjugated to each enzyme. The enzymes were mixed with NHS-Alexa488 at a molar ratio of 50 µM:150 µM in PBS (pH 7.4) and incubated at room temperature for 2 h. Following the labeling process, the excess dye was removed using PD-10 desalting columns (GE Healthcare, USA).

For quantification of the loaded enzymes, Alexa488-RDC/DAO@NC or RDC/Alexa488-DAO were placed in the upper chamber of the Nanosep^®^ centrifugal filter unit (MWCO 300 kDa). The filter device was centrifuged at 8,000 rpm for 40 min, followed by resuspension of the retained Alexa488-RDC/DAO@NC or RDC/Alexa488-DAO@NC in 400 μL of PBS. The fluorescence of the solution was then measured using a microplate reader (Varioskan Lux, Thermo Fisher, Waltham, MA, USA) to determine the amount of loaded enzyme, based on a standard curve of Alexa488-RDC or Alexa488-DAO.

To evaluate the release profile of RDC and DAO from the NC, Alexa488-RDC/DAO@NC was placed in the upper chamber of the Nanosep^®^ centrifugal filter unit (MWCO 300 kDa). The amount of RDC released from the NC was quantified by measuring the fluorescence signal of Alexa488, while the amount of released DAO was determined based on its enzymatic activity, as explained in the following section, ‘*Enzymatic activity assay of DAO*’. The filter unit was centrifuged at 8,000 rpm for 40 min at predetermined time points (0.04, 0.5, 1, 2, 3, and 5 days). After each centrifugation, the upper chamber was replenished with fresh release buffer, consisting of PBS (pH 7.4) supplemented with 0.1% bovine serum albumin (BSA) and 0.05% sodium azide (NaN_3_). The measurements of released RDC and DAO were conducted at each time point to determine their respective release kinetics.

**S3.3 Enzymatic activity assay of RDC**

The enzymatic activity of RDC was evaluated by measuring agmatine production at pH levels of either 6.4 or 7.4. The concentration of RDC was 0.1 mg/mL, and the reaction mixture included 0.2 mM pyridoxal-5′-phosphate (PLP). Arginine concentrations of 100, 50, 25, 12.5, and 6.25 mM were tested. Reactions (300 µL total volume) were incubated at 37 ℃ for 1 h. After incubation, agmatine concentration was determined using a butanol extraction assay [34]. 300 µL of 10% NaOH-saturated KOH solution and 200 µL of n-butanol were added to the reaction mixture. After vortexing and centrifugation, the upper butanol layer was mixed with 100 µL of diacetyl reagent, vortexed for 10 min, and centrifuged. The absorbance of the clarified supernatant was measured at 530 nm. A standard curve of agmatine was used for calibration.

**S3.4 Enzymatic activity assay of DAO**

The activity of DAO was determined at a concentration of 4 mg/mL, with the pH set to either 6.4 or 7.4. Various concentrations of the substrate agmatine (10, 1, 0.1, 0.01, and 0.001 mM) were tested to assess the enzymatic activity. Reaction volumes of 100 µL were incubated at 37 ℃ for 1 h. The production of hydrogen peroxide, a by-product of the DAO-catalyzed agmatine conversion, was quantified using a fluorescence detection method based on the horseradish peroxidase (HRP)/homovanilic acid (HVA) system. To this end, 100 µL of the reaction mixture was mixed with 2 units/mL HRP and 400 µM HVA, and the mixture was incubated for 2 min at room temperature. Following the incubation, the fluorescence was measured with a microplate reader at excitation/emission wavelengths of 321 nm/421 nm. The concentration of hydrogen peroxide generated during the reaction was quantified using a calibration curve of hydrogen peroxide.

|  | NC | RDC/DAO@NC |
| --- | --- | --- |
| Diameter (nm) | 122 ± 26 | 138 ± 17 |
| PDI | 0.334 ± 0.115 | 0.299 ± 0.053 |
| Zeta potential (mV) | 6.93 ± 1.69 | -2.31 ± 0.80 |

Table S1. Size and zeta potential of NC and RDC/DAO@NC at 37℃ measured by DLS.

**
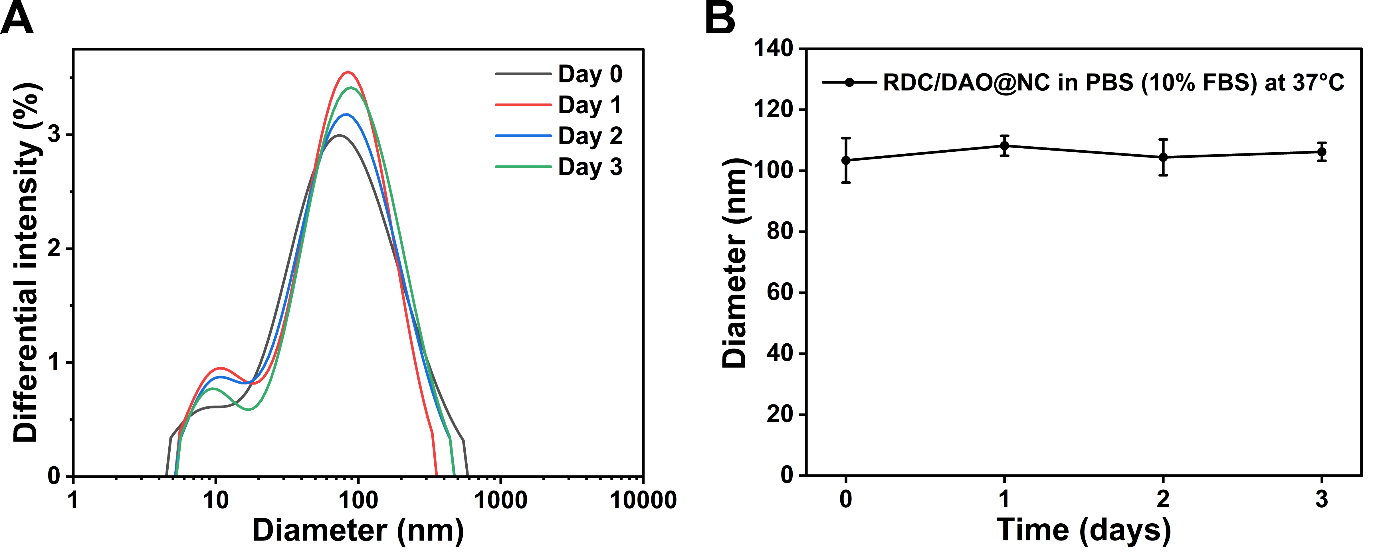
**

**Figure S5.** (A) Time-dependent DLS size-distribution profiles of RDC/DAO@NC incubated in PBS containing 10% FBS at 37 ℃ for 0–3 days. The small peak near ~10 nm is likely due to serum components. (B) Mean hydrodynamic diameter of RDC/DAO@NC during incubation in PBS containing 10% FBS at 37 ℃ for 0–3 days.

**
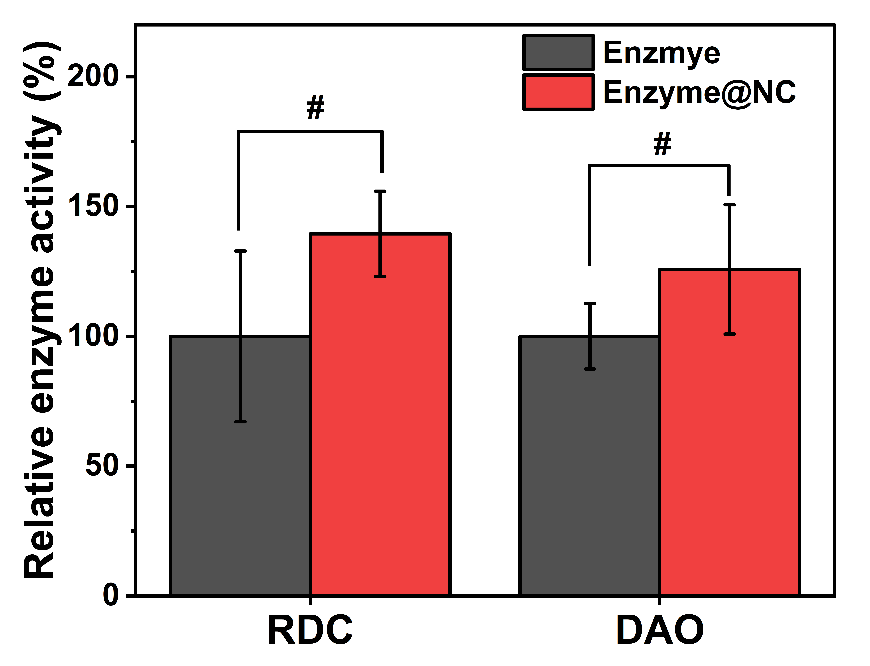
**

**Figure S6.** Comparison of enzyme activity before and after loading into NC (n=3). Each enzyme (RDC or DAO) was individually loaded into NC, and catalytic activity was measured at pH 6.4. No significant difference was observed between free enzymes and NC-encapsulated enzymes. Statistical significance: #p>0.05.

**
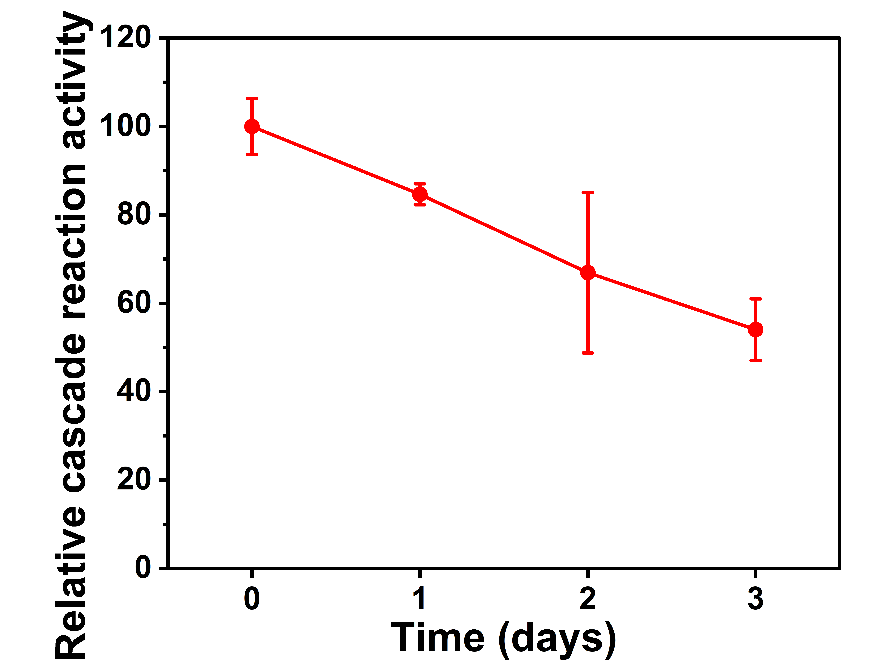
**

**Figure S7.** Time-dependent cascade reaction activity of RDC/DAO-loaded NC at pH 6.4 and 37 ℃ (n=3).

**S4. ASS1 mRNA expression analysis in SCC7 cells**

To quantify ASS1 mRNA expression in SCC7 cells, Raw264.7 cells, used as a positive control, and SCC7 cells were each seeded in 6-well plates at a density of 10^6^ cells per well. Raw264.7 cells were divided into two groups: one treated with lipopolysaccharide (LPS, 500 ng/mL) and one left untreated. After 24 h of incubation, total RNA was isolated using Tri-RNA reagent, and cDNA was synthesized using the High-Capacity cDNA reverse transcription kit from the extracted RNA according to the manufacturer’s protocol. qRT-PCR was subsequently performed using SYBR Green reagent with mouse GAPDH (forward: 5’-AGGTCGGTGTGAACGGATTTG-3’, reverse: 5’-TGTAGACCATGTAGTTGAGGTCA-3’) and mouse ASS1 primers (forward: 5’-CACTCTACGAGGACCGCTATCT-3’, reverse: 5’CTCAAAGCGGACCTGGTCATTC-3’).

**
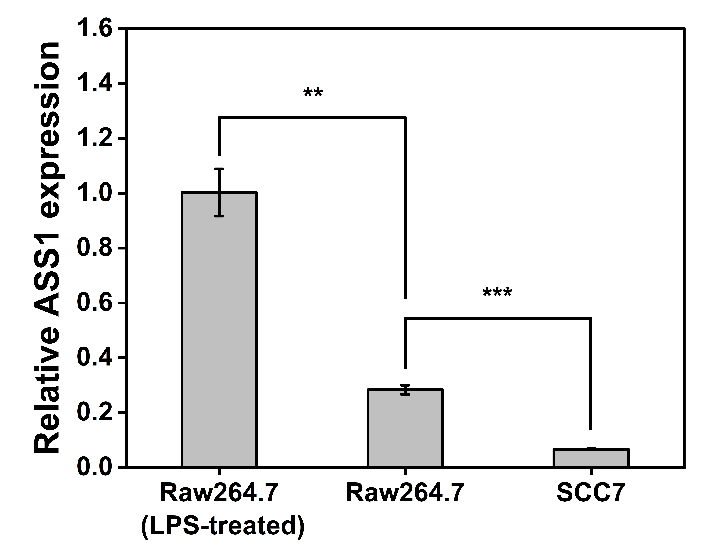
**

**Figure S8.** Relative ASS1 mRNA expression measured by SYBR Green-based RT-qPCR. LPS-stimulated Raw264.7 cells (500 ng/mL) were included as an ASS1-positive control (n=3). Statistical significance: #p>0.05, *p<0.05, **p<0.01, ***p<0.001.

**S5. *In vitro* antitumor assay**

**
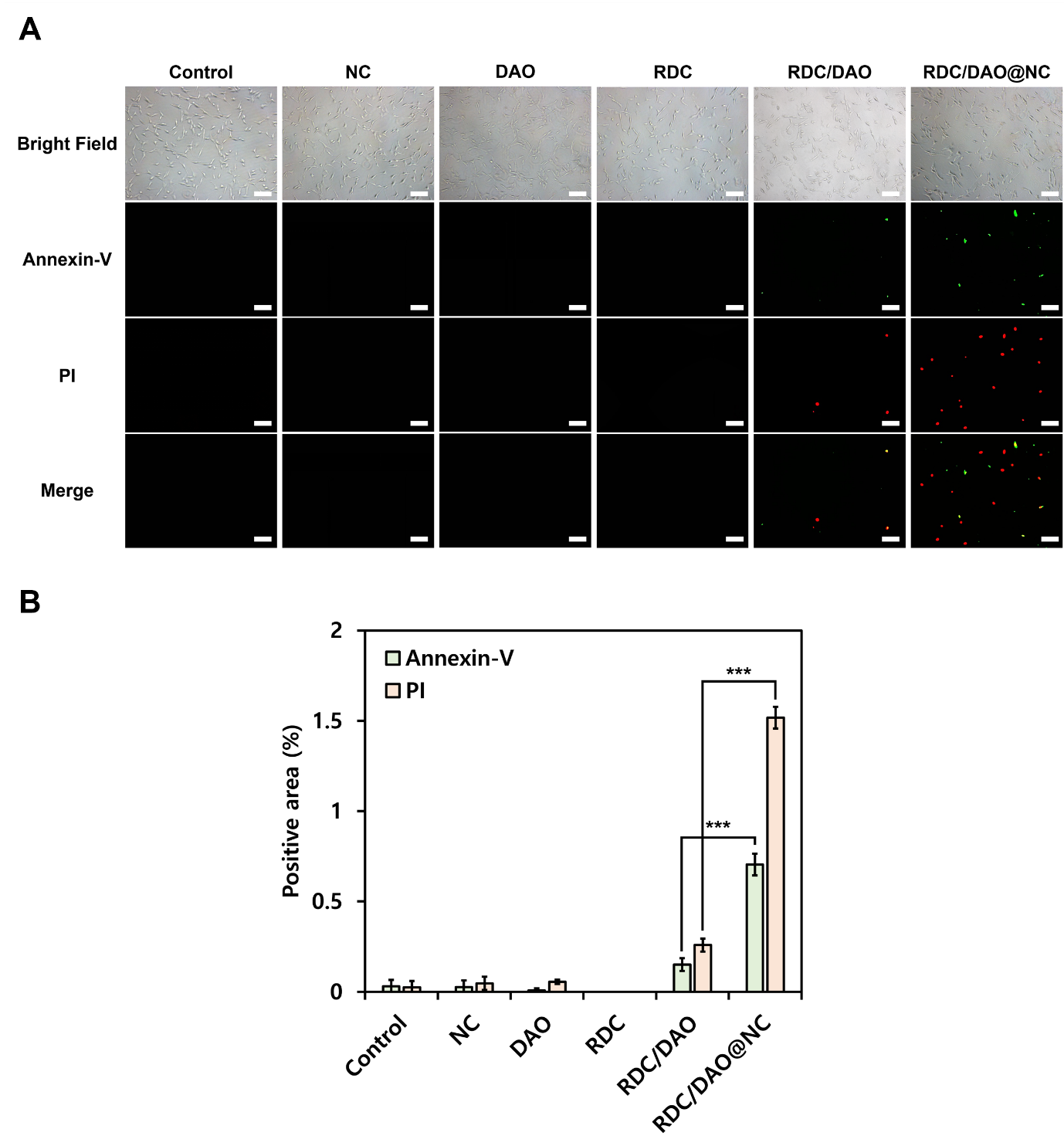
**

**Figure S9.** *In vitro* apoptosis analysis of SCC7 cells using Alexa Fluor 488-Annexin V and PI staining. (A) Representative Alexa Fluor488-Annexin V and PI staining images of SCC7 cells treated with NC (2.5 mg/mL), RDC (1 mg/mL), or DAO (1 mg/mL) for 24 h. Scale bar = 100 μm. (B) Quantification of Annexin V-positive and PI-positive cells (n=5). Statistical significance: #p>0.05, *p<0.05, **p<0.01, ***p<0.001.

**
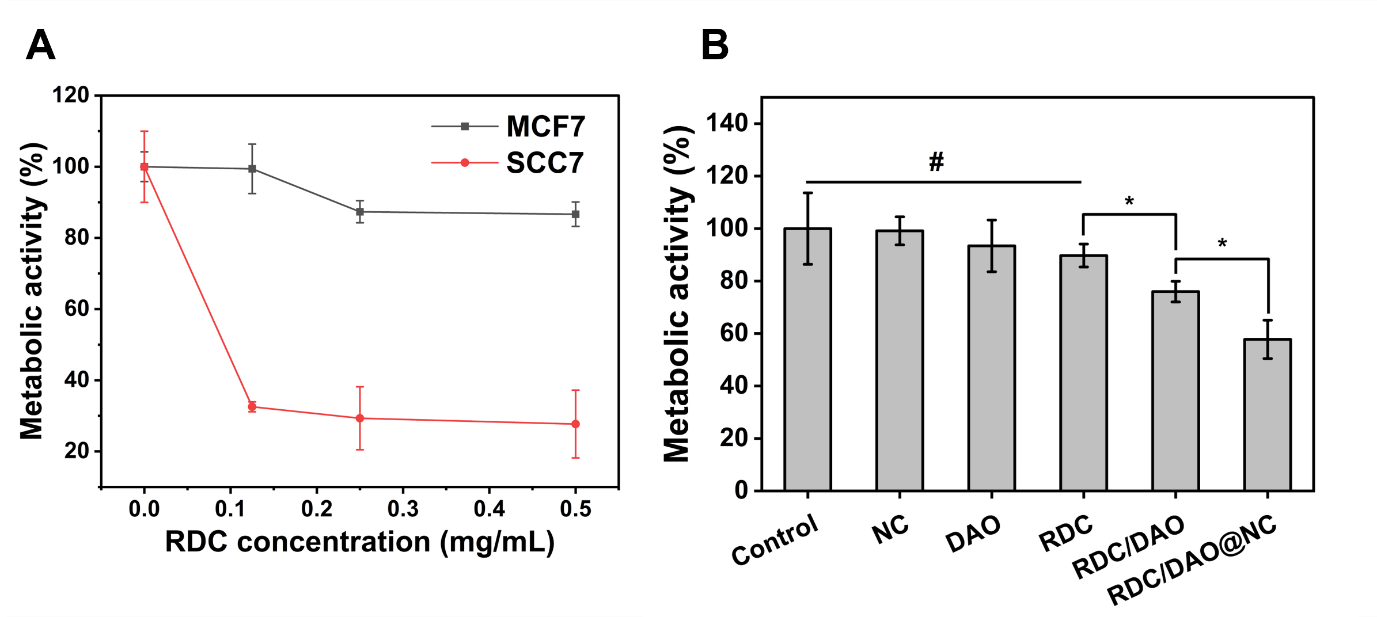
**

**Figure S10.** (A) Metabolic activity of MCF7 and SCC7 cells following 72 h incubation with increasing concentration of RDC under mildly acidic conditions (pH 6.4) (n=3). (B) Metabolic activity of MCF7 cells following 72 h incubation with NC, RDC, DAO, RDC/DAO, and RDC/DAO@NC (RDC: 0.1 mg/mL; DAO: 0.1 mg/mL; NC: 0.25 mg/mL) under mildly acidic conditions (pH 6.4) (n=3). Statistical significance: #p>0.05, *p<0.05.

**S6. *In vivo* analysis**

**
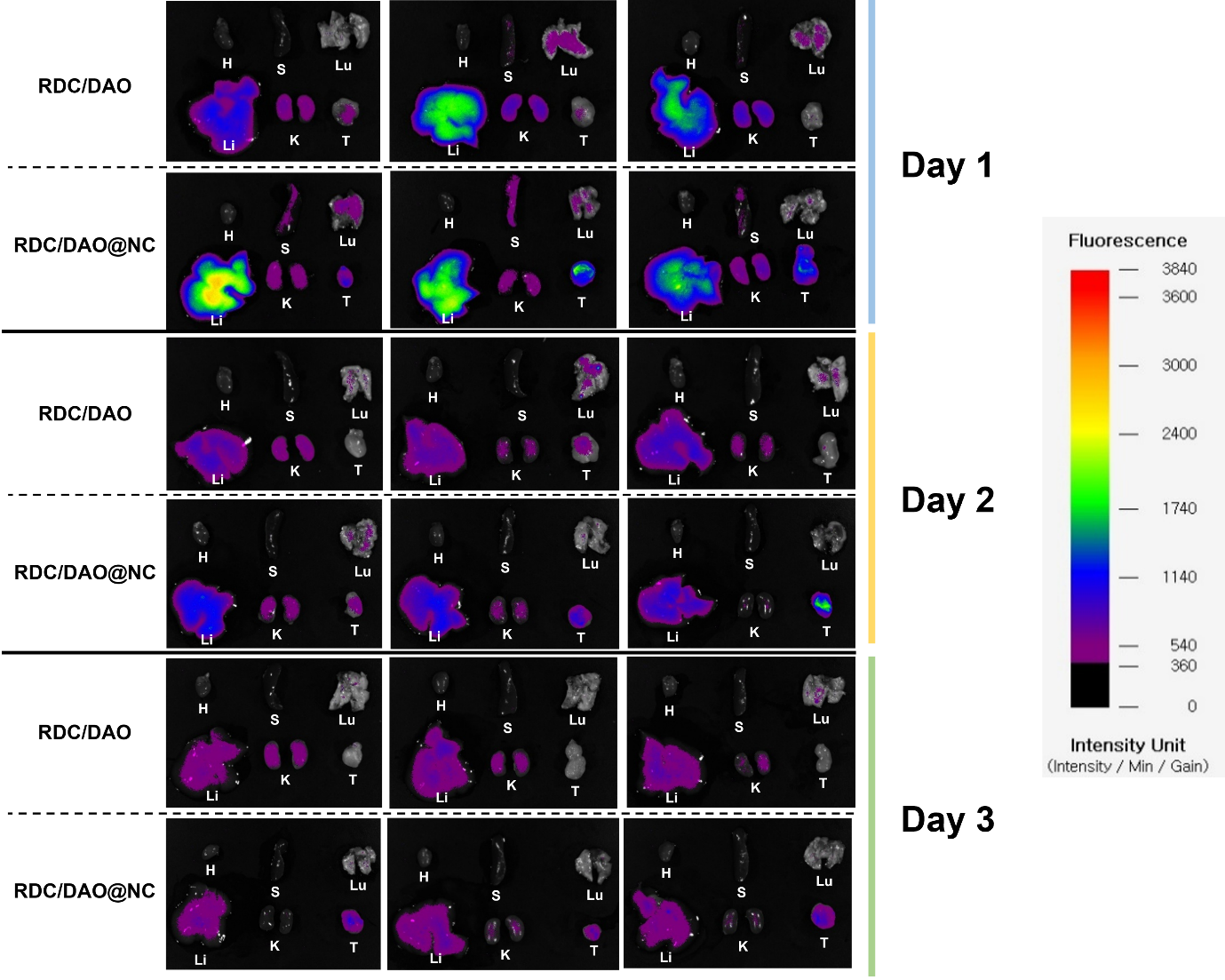
**

**Figure S11.** Total e*x vivo* fluorescence images of major organs and tumor tissues collected from SCC7 tumor-bearing mice at designated time points after intravenous injection of Alexa680-RDC/DAO or Alexa680-RDC/DAO@NC (H: heart, S: spleen, Lu: lung, Li: liver, K: kidney, T: tumor).

***
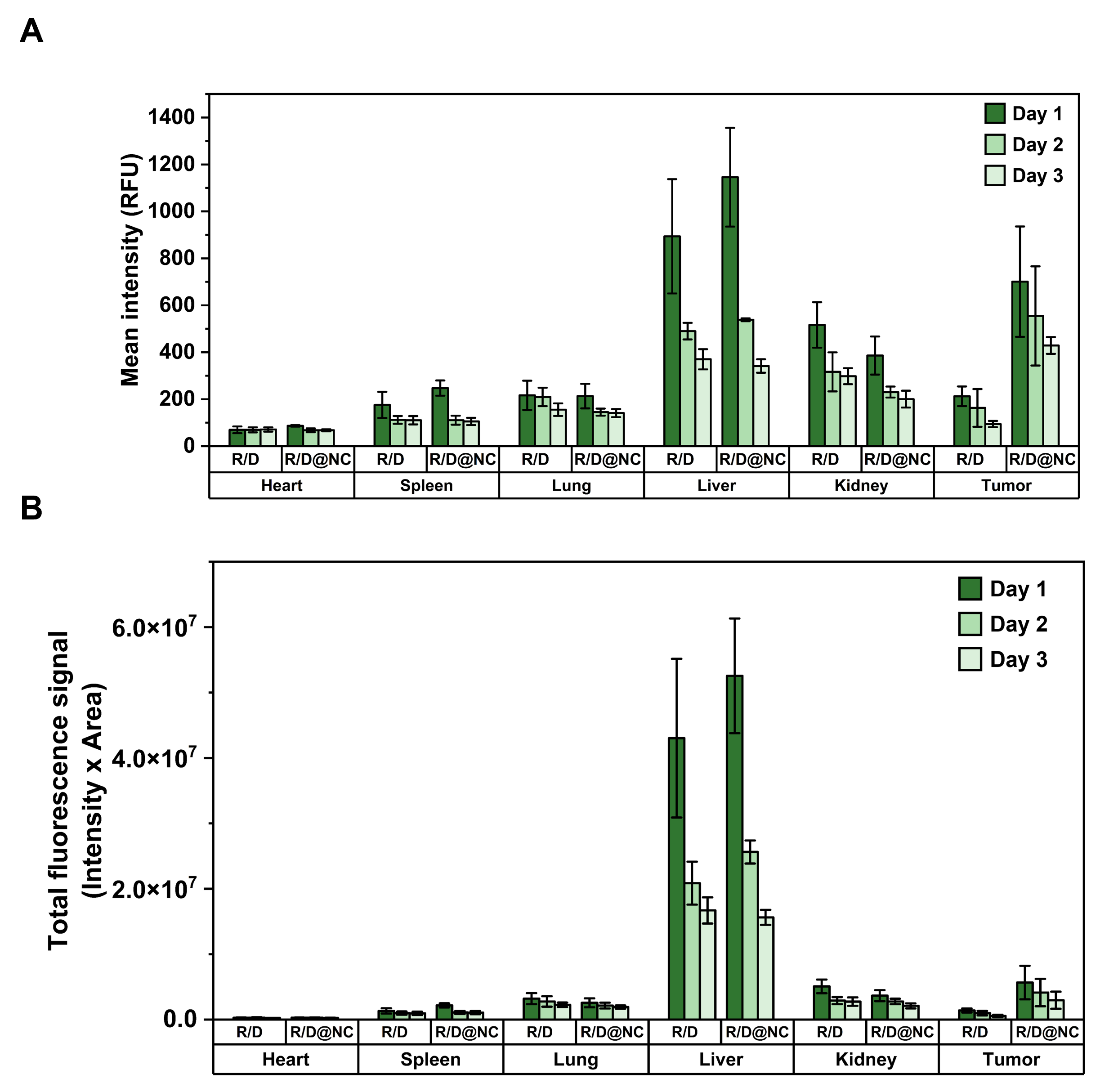
***

**Figure S12**. Quantitative biodistribution analysis of Alexa680-labeled RDC/DAO and RDC/DAO@NC in major organs and tumors. (a) Mean fluorescence intensity of Alexa680 signals measured in excised organs (heart, spleen, lung, liver, kidney) and tumor tissues at Days 1, 2, and 3 post-injection, expressed as relative fluorescence units (RFU). (b) Total fluorescence signal calculated as ‘mean fluorescence intensity (RFU) × tissue area (pixels)’.

**
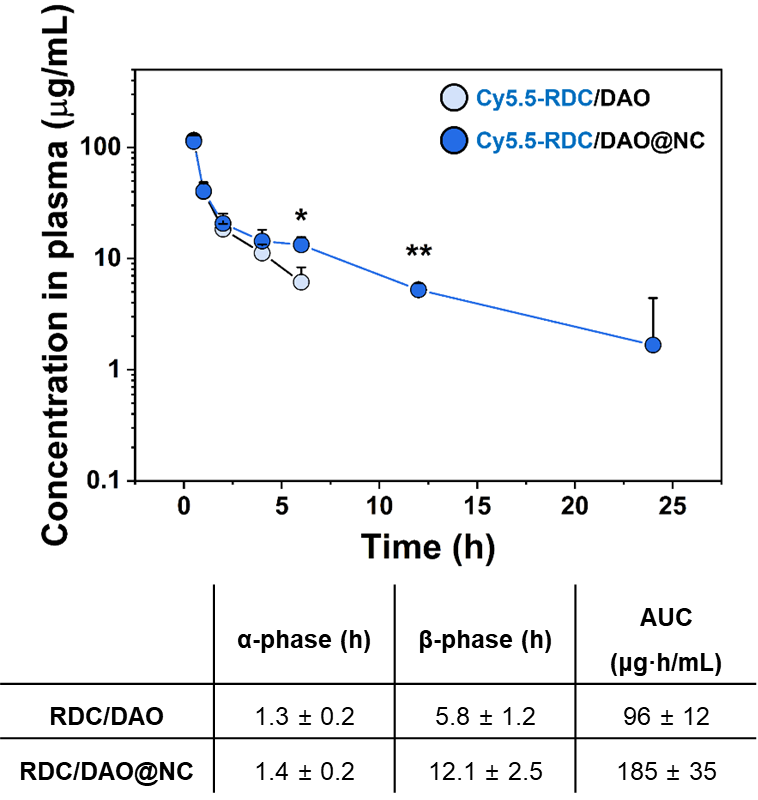
**

**Figure S13**. Plasma pharmacokinetics of Cy5.5-labeled RDC/DAO and RDC/DAO@NC following intravenous injection. Blood samples were collected at 0.5, 1, 2, 4, 6, 12, and 24 h after injection (n=3). The plasma concentration of Cy5.5-RDC was determined based on fluorescence intensity, and pharmacokinetic analysis was conducted using RDC as the representative marker. Cy5.5-RDC/DAO was not detectable after 12 h (detection limit: 0.1 μg/mL). Quantitative pharmacokinetic parameters, including circulation half-life (t₁/₂,α and t₁/₂,β) and AUC, are summarized alongside the graph. The α-phase was defined over 0–2 h, while the β-phase was determined over 2–6 h for RDC/DAO and 2–24 h for RDC/DAO@NC. Statistical significance: #p > 0.05, *p < 0.05, **p < 0.01.

**
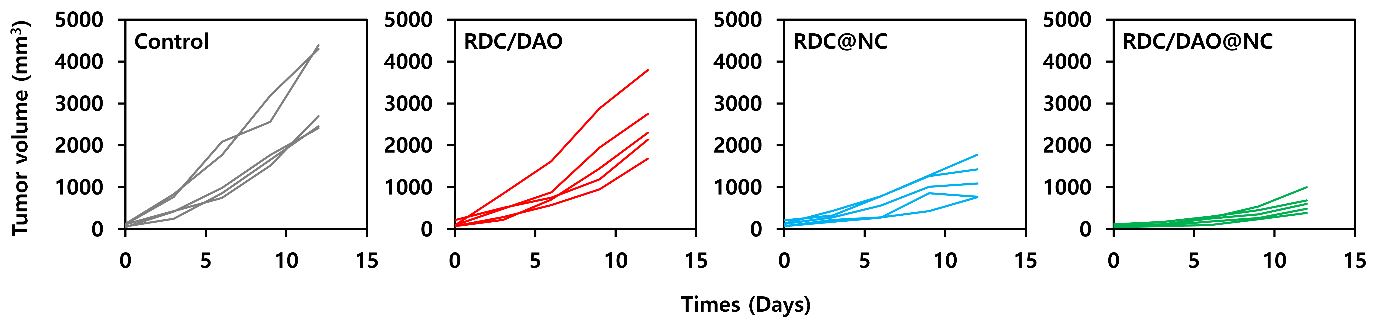
**

**Figure S14**. Tumor growth curves for all treatment groups. Tumor growth was monitored in SCC7 tumor-bearing mice treated with different formulations, including PBS (control), RDC/DAO, RDC@NC, and RDC/DAO@NC (n=5). Tumor volumes were measured every 3 days.

**
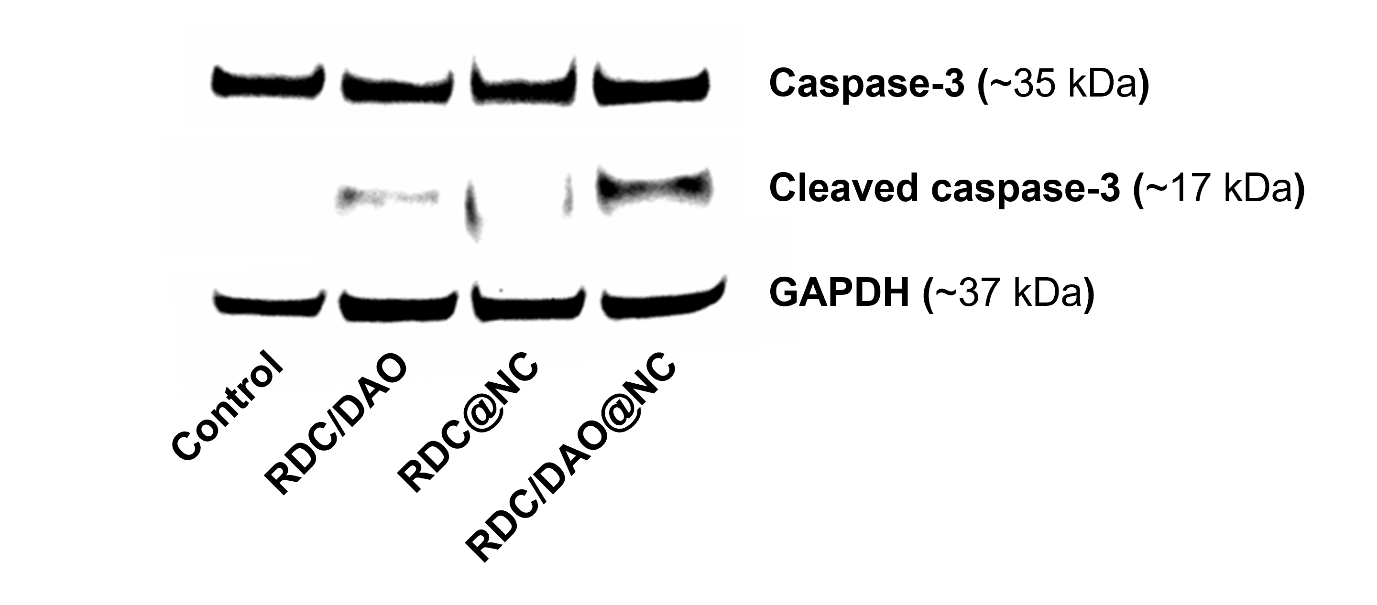
**

**Figure S15**. Western blot analysis of tumor tissue lysates showing caspase-3 (~35 kDa), cleaved caspase-3 (~17 kDa), and GAPDH (~37 kDa) expression in each treatment group (Control, RDC/DAO, RDC@NC, RDC/DAO@NC).

**
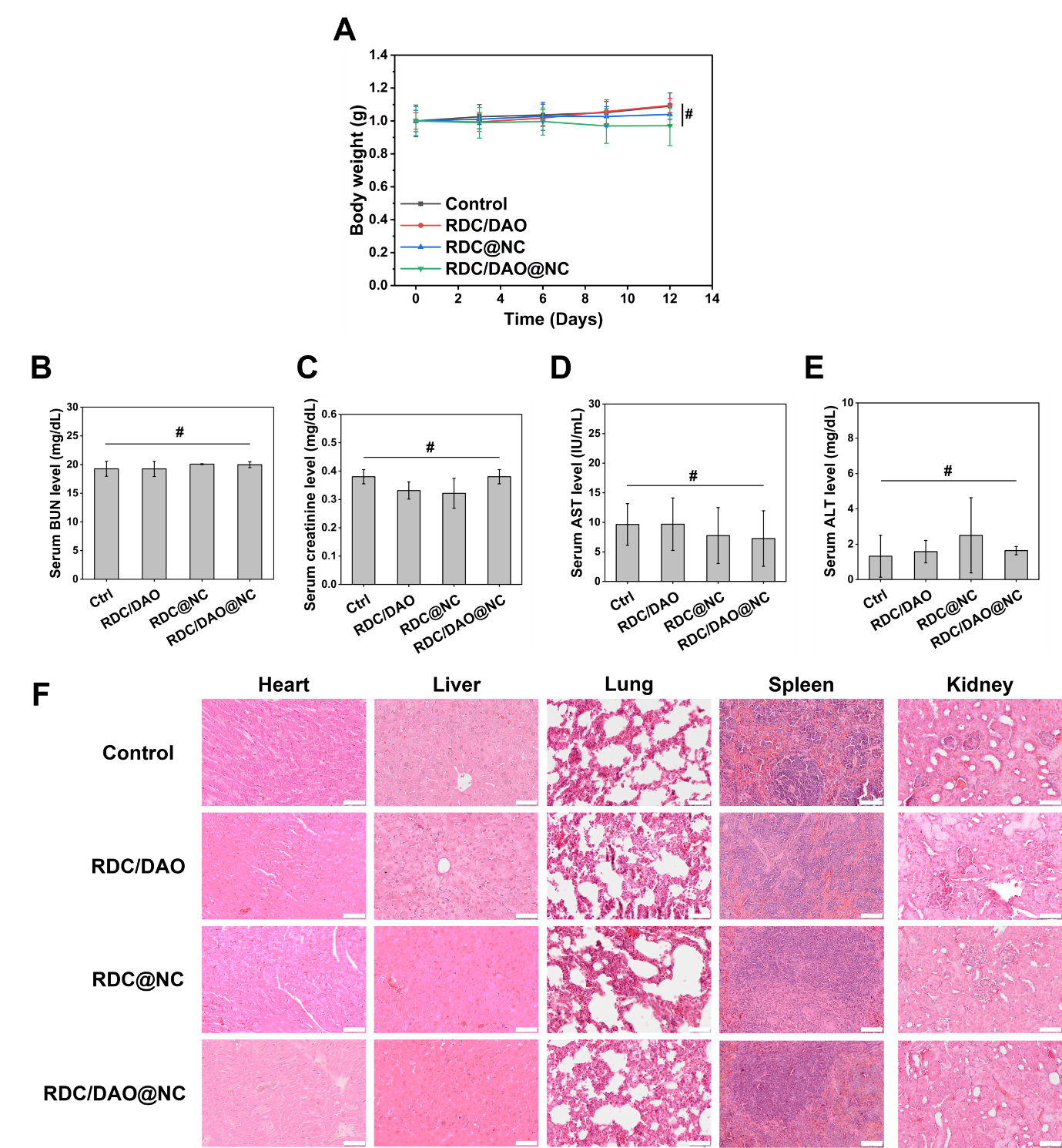
**

**Figure S16**. *In vivo* biocompatibility assessment of RDC/DAO@NC. (A) Body weight monitoring of mice treated with PBS (control), RDC/DAO, RDC@NC, or RDC/DAO@NC over 12 days (n=5). Body weights were measured every 3 days. Serum biochemical analysis of kidney and liver function, including (B) blood urea nitrogen (BUN), (C) creatinine, (D) aspartate aminotransferase (AST), and (E) alanine aminotransferase (ALT), after treatment with the indicated samples (n=5). (F) Representative H&E staining images of major organs (heart, liver, lungs, spleen, and kidneys) from mice treated with the indicated formulations. Scale bar = 100 μm. Statistical significance: #p>0.05, *p<0.05, **p<0.01.
